# ANN-GA based biosorption of As(III) from water through chemo-tailored and iron impregnated fungal biofilter system

**DOI:** 10.1038/s41598-022-14802-w

**Published:** 2022-07-20

**Authors:** A. Tripathi, M. R. Ranjan, D. K. Verma, Y. Singh, S. K. Shukla, Vishnu D. Rajput, Tatiana Minkina, P. K. Mishra, M. C. Garg

**Affiliations:** 1 Amity Institute of Environmental Sciences, Amity University Uttar Pradesh, Noida-125, Gautam Buddha Nagar, U.P. 201303 India; 2 School of Biochemical Engineering, Indian Institute of Technology (Banaras Hindu University), Varanasi, U.P. 221005 India; 3 Department of Transport Science and Technology, School of Engineering and Technology, Central University of Jharkhand, Ranchi, Jharkhand 835222 India; 4 Academy of Biology and Biotechnology, Southern Federal University, Rostov-on-Don, Russia 344090; 5 Department of Chemical Engineering, IIT BHU, Varanasi, U.P. 221005 India

**Keywords:** Chemical biology, Environmental sciences

## Abstract

The iron impregnated fungal bio-filter (IIFB) discs of luffa sponge containing *Phanerochaete chrysosporium* mycelia have been used for the removal of As(III) from water. Two different forms of same biomass viz. free fungal biomass (FFB) and modified free fungal biomass (chemically modified and iron impregnated; CFB and IIFB) have been simultaneously investigated to compare the performance of immobilization, chemo-tailoring and iron impregnation for remediation of As(III). IIFB showed highest uptake capacity and percentage removal of As(III), 1.32 mg/g and 92.4% respectively among FFB, CFB and IIFB. Further, the application of RSM and ANN-GA based mathematical model showed a substantial increase in removal i.e. 99.2% of As(III) was filtered out from water at optimised conditions i.e. biomass dose 0.72 g/L, pH 7.31, temperature 42 °C, and initial As(III) concentration 1.1 mg/L. Isotherm, kinetic and thermodynamic studies proved that the process followed monolayer sorption pattern in spontaneous and endothermic way through pseudo-second order kinetic pathway. Continuous mode of As(III) removal in IIFB packed bed bioreactor, revealed increased removal of As(III) from 76.40 to 88.23% with increased column height from 5 to 25 cm whereas the removal decreased from 88.23 to 69.45% while increasing flow rate from 1.66 to 8.30 mL/min. Moreover, the IIFB discs was regenerated by using 10% NaOH as eluting agent and evaluated for As(III) removal for four sorption–desorption cycles, showing slight decrease of their efficiency by 1–2%. SEM–EDX, pHzpc, and FTIR analysis, revealed the involvement of hydroxyl and amino surface groups following a non-electrostatic legend exchange sorption mechanism during removal of As(III).

## Introduction

Arsenic contamination of groundwater is a serious global environmental concern affecting a large number of populations, especially in developing countries. Chronic arsenic toxicity causes severe consequences to human health, such as skin lesions, circulatory and nervous disorders, black foot disease, melanosis, keratosis, non pitting oedema, Bowen’s disease gangrene and cancers of different organs^1,2^. Thus, the removal of arsenic from potable water is an essential requirement.

In India, as per the reports of CGWB, GOI, in year 2009, seven states namely, West-Bengal, Jharkhand, Bihar, Uttar Pradesh in the flood plain of Ganga River; Assam and Manipur in the flood plain of Brahamaputra and Imphal rivers and Rajnandgaon village in Chhattisgarh state have so far been reported affected by arsenic contamination in groundwater above the permissible limit of 50 µg/L. At certain location tube wells were found to have Arsenic concentration of 300 µg/L. The present study confirms treatment of more than highest concentration of Arsenic observed in groundwater samples. People in these affected states have chronically been exposed to arsenic drinking arsenic contaminated hand tube-wells water. With every new survey, more arsenic affected villages and people suffering from arsenic related diseases are being reported, and the problem resolving issues are getting complicated by a number of unknown factors. It is now generally accepted that the source is of geological origin and percolation of fertilizer residues may have played a modifying role in its further exaggeration.

The two major approaches are recently being utilized by the researchers to develop the advanced tertiary treatment systems for improving the quality of waste water as well as potable water. First approach is focused on physico-chemical treatment processes viz*.* adsorption, coagulation, filtration, osmosis, redox reaction, ion exchange, electrodialysis and photo catalysis^3–13^. However, the second approach follows the application of living system or their dead biomass for removal of contaminants through biofiltration, bioremediation and biosorption^5,14,15^. Both the treatment sectors have its own advantages and drawbacks, such as cost-intensiveness, complexity and non-eco-friendly nature in case of physico-chemical treatment processes, however biosystems are hard to maintain having poor mechanical stability and excretory metabolic byproducts.

Being inspired from a biofilter system, an attempt has been made in the current study to develop an efficient biosorptive filtration system by merging the properties of simplicity and robustness of physico-chemical systems along with economic viability and eco-friendly nature of biological systems. By definition, any type of filter having attached biomass on the filter-media can be defined as a biofilter^16^. The basic difference between a conventional filter and biofilter is that the conventional filters remove pollutants due to physical straining, whereas biofilters eliminate the pollutants through metabolic activities of living microorganisms. The organic material present as pollutant is utilized as nutrient by growing organisms of a biofilter system. But in our case, the targeted contaminant [As(III)] is mainly present in source drinking water, which lack organic materials to support the microbial growth. Thus, the application of a conventional biofilter for concerned purpose is not feasible. Additionally, the safety of water treated through biofilter might not be assured for drinking in a long run due to probable presence of harmful metabolites, pathogenic spores and toxic byproducts of degraded pollutants produced by microbes. Thus, developing a sorption based biofilter by using dead biomass can be a good approach against the limitations of a conventional biofilter. Unlike living cells, the dead biomasses can act as excellent sorbent as they are easy to handle, require no nutritional supply and provide higher flexibility for chemical modification^5^. Huge amount of literature is available on chemical modification of different biomasses for the improvement of its adsorption capacity^17^.

The present study demonstrates a novel approach of dealing with development and evaluation of sorption-based filtration system using modified and immobilized dead biomass of fungus *Phanerochaete chrysosporium,* over plant derived 3D matrix of cellulose fibres (luffa sponge). Luffa sponge is an inexpensive and easily available natural material having open and interwoven network of cellulosic fibers obtained from ripen fruits of *Luffa cylindrica* plant. Luffa sponge is not only an immobilizing material, but also allows rapid and less compact mycelial growth over it. Immobilization due to growth of fungus over luffa sponge minimized diffusion barrier problem which occurs in conventional immobilization matrixes. Along with this the limitations of mechanical strength and reusability of microbial biomass can also be overcome by such immobilization approach^18^.

An important step in biosorptive systems is to achieve maximum efficacy by optimizing the process parameters through manual methods or by applying computational approaches. However, vast experimentation is required to screen the optimized parameters in conventional optimization method which is much laborious, time consuming and adds extra cost. Further, a synergistic approach of response surface methodology (RSM) and artificial neural network—genetic algorithm (ANN-GA) were implemented to conclude the preciseness of operational parameters^19,20^.

## Materials and methods

Fungus *Phanerochaete chrysosporium* MTCC 4955 was purchased from IMTECH, Chandigarh, India and the maintenance and growth media included chemicals: malt extract, peptone, glucose, agar; d-glucose, MgSO_4_.7H_2_O, KH_2_PO_4_, NH_4_Cl, CaCl_2_ 0.2H_2_O. Thiamine respectively. Other chemicals used were-Anhydrous Sodium arsenite (NaAsO2), Iron chloride Hexahydrate (FeCl3. 6H2O), Sodium hydroxide (NaOH), Hydrochloric acid (HCl). In whole experiments deionized water was used for stock and fresh solution. All chemicals and reagents used were of analytical grade and were employed without further purification (purchased from Himedia, Mumbai, India). Luffa sponge, a natural material having open and interwoven network of cellulosic fibres obtained from ripen fruits of *Luffa cylindrica* plant was obtained from a farmer nearby Banaras Hindu University, Varanasi, Uttar Pradesh, India. All glassware used in this work was rinsed with 10% nitric acid (suprapure, 69%) to remove all impurities that might be present and to prevent further adsorption of heavy metals to the walls of the glassware.

### Microorganism and culture medium

A white rot fungus, *Phanerochaete chrysosporium* MTCC 4955, was maintained and stored on malt extract agar (MEA) slants containing (g/L): malt extract 20.0; peptone 1.0; glucose 20.0 and 20.0 (pH 5.5). While, the growth media of the microorganism consisted of the following ingredients: (g/L): D-glucose 10.0; MgSO_4_.7H_2_O 0.5; KH_2_PO_4_ 2.0; NH_4_Cl 0.1; CaCl_2_ 0.2H_2_O 0.1; Thiamine 0.001 at pH 4.5^21^.

### Preparation of free fungal biomass, chemo tailored fungal biomass and immobilized and iron impregnated fungal biomass (FFB, CFB and IIFB)

Luffa Sponge obtained from a farmer nearby Banaras Hindu University, Varanasi, Uttar Pradesh, India was used as immobilization matrix. Luffa sponge was cut into circular discs of 2 cm in diameter and 2 mm in thickness. Followed by cutting circular discs were soaked in boiling water for 30 min, thoroughly washed under tap water, soaked for 24 h and rinsed for 3 to 4 times with deionized water. Then, the sponge discs were sterilized by autoclaving at 121 °C for 30 min. Finally, the sponge discs were oven dried at 70 °C and stored in desiccators before further use. The fungal spore suspension was prepared by suspending the fungal spores (15 days old) in 10 mL of sterile water. Thereafter, two sets each with 10 flasks (250 mL) were arranged. First set of flasks was containing 100 mL of growth media while second set of flaks was containing 100 mL of growth media along with 5 prior washed luffa sponge discs of 2 cm in diameter and 2 mm in thickness. The media of each flask in both sets was inoculated with 0.5 mL of spore suspension followed by incubation at 25 °C and 100 rpm in an incubator shaker for 8 days. A schematic flow-sheet describing comprehensive methodology for the production of different forms of biomass was provided as supplementary material (Supplementary data: Figure S1). After 8 days, the flasks of set one produced the free fungal biomass (FFB), which was harvested through filtration. Whereas second set of flasks produced immobilized fungal biomass (IFB) over luffa sponge discs (*step 1*). Immobilization of the fungus onto luffa sponge has been performed according to our previous study^21^. FFB and IFB produced in *step 1* were used for examining the effect of immobilization by comparing their efficiencies for As(III) removal.

In *step 2,* dried FFB and IFB were chemo-tailored by treating them with 0.1 N HCl. For chemical modification, 5 g of both the dried biomasses were individually suspended in 100 mL of 0.1 N HCl and incubated at the agitation condition of 100 rpm at 60 °C for 6 h. The resulting chemo-tailored FFB and IFB biomasses were collected and washed thoroughly with deionised double distilled water till pH of rinsed water was stabilized. The effect of chemo-tailoring on As(III) removal efficiency of biomasses was again analysed by keeping FFB as control. The chemo-tailored biomasses were modified through iron impregnation at pH range from1 to 9 as in *step 3* in order to further improve the removal capacity of biomasses following the method described by^22^. Iron impregnation approach has been well implemented to enhance the sorption capacity and affiliated removal efficiency for As(III) in different studies^22,23^. After iron impregnation, chemo-tailored FFB provides chemo-tailored and iron-impregnated free fungal biomass (CFB) while chemo-tailored IFB yields immobilized and iron-impregnated fungal biomass (IIFB). The amount of impregnated iron per unit mass of CFB and IIFB was quantified by acid digestion method (U.S. EPA 3050B). Further, the As(III) removal was studied using these two biomasses (i.e. CFB and IIFB) by keeping FFB as control to comprehend the parallel effect of all the modifications i.e. immobilization, chemo-tailoring and iron impregnation.

### Batch biosorption studies in shake flasks

The parameters affecting biosorption over the biomass viz*.* pH, biomass dose, initial metal concentration, temperature and contact time were investigated by conducting individual biosorption experiments^24^. Preliminary experiments were conducted with FFB, CFB and IIFB in order to get the optimal values of aforesaid parameters, selecting them arbitrarily by following one factor at a time approach, in the range of pH 2 to 10, biomass dose 0.1 to 1.0 g/L, temperature 20 to 50 °C and metal ion concentration 0.1 to 2.0 mg/L.

However, all the experiments were re-performed by varying one factor, while keeping others at their optima for examining real behavior of a parameter in relation to others, reproducibility of results and actual difference in performances of FFB, CFB and IIFB for As(III) removal. The final results, thus obtained, have been taken in to account and are discussed here. Experiments were performed in 250 mL conical flasks along with 100 mL of As(III) solution and desired amount of biomass followed by incubation under agitating condition at 100 rpm in rotary shaking incubator until the establishment of equilibrium. Samples were examined at regular time interval to evaluate the concentration of As(III) in the solution. All the experiments were conducted in triplicates and average values were taken for the data analysis.

In order to justify the non-electrostatic exchange mechanism during sorption of As(III), the zero point charge of FFB and IIFB was measured with the help of solid addition method discussed by^25^.

### Bio-filtration studies in up-flow packed bed bioreactor

Bio-filtration experiments in continuous mode were carried out in borosilicate glass bioreactor (diameter 2 cm, length 30 cm). IIFB discs were stacked between two supporting layers of glass wool of thickness 1 cm to attain certain bed heights. Approximately 2 cm length of bioreactor was packed with glass beads on both sides of glass wool for dispersing the flow and holding the glass wool in position. Metal solution of different initial concentration was pumped in an up-flow mode within the bioreactor at desired flow rate using a peristaltic pump (Miclins PP-10). Extracted samples were analyzed for residual As(III) concentration at regular basis. After the exhaustion of bed, 250 mL of deionized double distilled water was passed through bioreactor to wash out the un-adsorbed metal from the bioreactor. For desorption of attached metal and recharging the column, 10% NaOH was passed as eluting agent in an up-flow mode through the bioreactor at a flow rate of 1.66 mL/min. The effluent solution was collected and analyzed for elution efficiency.

### Analysis of arsenic in aqueous solution

Arsenic analysis in the sample solution was conducted using atomic absorption spectrophotometer (AAS, Shimadzu AA-6300). In order to produce the hydrides of the metal ions, basic unit of continuous flow hydride vapor generator (HVG, Shimadzu HVG-1) was coupled to the AAS. In order to examine arsenic, the hollow cathode lamp was employed as sole light source, adjusted at 1937 Aº wavelength, 12 mA lamp-current and 7 Aº slit width with deuterium lamp for background correction. For the generation of flame for the AAS instrument grade (98%) acetylene was supplied at 4.0 L/min at a pressure of 0.9 kg/cm^2^, along with compressed air delivered with a flow rate of 17.5 L/min and gas pressure of 3.5 kg/cm^2^. High purity (99.99%) argon was used as purge gas at a 70.0 mL/min flow rate and 3.2 ± 0.2 kg/cm^2^ supply pressure.

### Optimization of adsorption parameters

#### Response surface design (RSM)

RSM is a common statistical tool for building quadratic models for providing the statistical relationship among the variables and determining the optimal process conditions. The four process variables viz*.* biomass dose, pH, temperature and initial As(III) concentration were chosen for experimental design to optimize As(III) biosorption by applying a technique under RSM i.e. central composite design (CCD). A full factorial CCD was applied to create a set of 31 experiments with the help of statistical software MINITAB version 17, USA. The results obtained from each individual experiment were adopted for performing regression analysis and ANOVA (analysis of variance) testing. A polynomial equation comprising a cumulative response (*Y*) with linear, square and linear interactive steps can be represented as below (Eq. 1):

1 $$Y = \beta_{0} + \sum_{i = 1}^{n} {\beta_{i} X_{i} } + \sum_{i = 1}^{n} {\beta_{ii} X_{i}^{2} } + \sum_{i = 1}^{n} {\sum_{j = 1}^{n} {\beta_{ij} X_{i} } } X_{j}$$

where β_*0*_ is the constant, n represents the number of variables, *β*_*i*_ is the slope or linear effect of the input variable *X*_*i*_, *β*_*ii*_ denotes the quadratic effect of input factor *X*_*i*_ and *βij* stands for linear by linear interaction effect between the input variable *X*_*i*_ and *X*_*j*_.

#### Artificial neural network (ANN)

**Figure 1:**
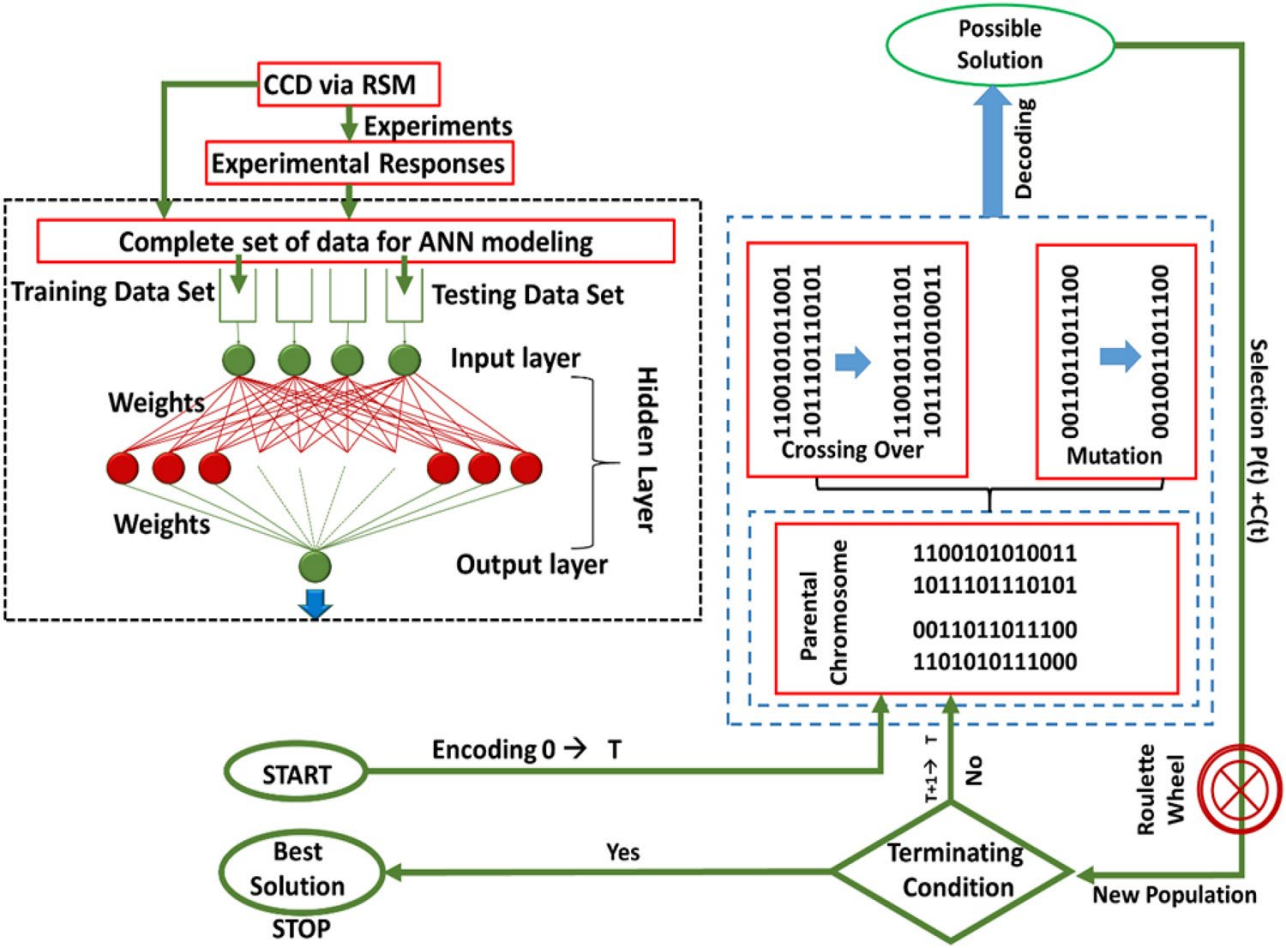
Schematic representation of simulated hybrid model (RSM-ANN-GA) and its working methodology.

ANN is experimentally proved advanced and more precise modeling technique in comparison to RSM as it comprises the nonlinearities of a fitness function significantly in a unique way^26^. Thus, a neural network with feed forward and back propagation architecture (FFBP) was employed by utilizing the experimental dataset generated through CCD to optimize the process parameters more precisely (Fig. 1). The MATLAB software (Version R2014b, Mathworks, Inc., USA) was applied for ANN-GA based modeling and data simulation. The topology for neural network was composed of three layers of neurons: an input, a hidden and an output layer. All these layers were interlinked together through a weighted connection in the forward direction. The experimental data of CCD was subdivided into two sets, training (24 experiments) and testing set (7 experiments). ANN algorithm was trained utilizing the training data, while the ANN performance was estimated on test data sets.

The input layer of the network just supplied input data to the nodes of hidden layer through some weights. In hidden layer, sum total weight of inputs was estimated as by the equation (Eq. 2):

2 $$Sum = \sum\limits_{i = 1}^{n} {x_{i} w_{i} + \theta }$$

Here *x*_*i*_ (*i* = 1, *n*) denotes for input dataset, *w*_*i*_ stands for the correlated weight and *θ* represents bias. Thereafter, the weighted output was further transmitted by hidden neurons through an activation function. Primarily weight and bias values were selected arbitrarily; but, in successive training steps, the weights and biases in hidden and output layers were synchronized with a convergence standard to obtain resemblance in training and testing experimental outcome. The logistic output function used in this simulation can be shown by the following equation (Eq. 3):

3 $$f(Sum) = \frac{1}{{1 + \exp \left( { - Sum} \right)}}$$

The learning of the neural network was conducted initially until it approached to an optimal value of coefficient of correlation (*R*^2^). The goodness of fit of created model can be represented significantly by *R*^2^ value. The statistical difference between experimental and predicted values of parameters through computational model is further revealed by mean square error (MSE) values.

#### GA optimization

After building up a generalized form of feed forward ANN model, its output values (i.e. weights and bias) was optimized by genetic algorithm (GA) as the following output function (Eq. 4):

4 $${Y}_{output}={weight}^{o}\times \left(\frac{2}{1+{e}^{\left(-2\times {weight}^{H }\times inputventor \times outputbias\left({b}^{l}\right)\right)}}-1\right)+hiddenlayerbias({b}^{H})$$

where *weights*^*0*^ represents weight on connections between hidden and output layer. However, *weights*^*H*^ expresses the weight on connections between input and hidden layer. GA creates an initial population of arbitrarily built chromosomes (expressed as series of binary digits) and pursues the analogy of natural selection to resolve the non-linear equations^26^. Further, in order to produce new populations of chromosomes from pre-existing one, three genetic operations: selection, crossover and mutation were introduced (Fig. 1). Selection operator facilitates to pick the best chromosomes as breeding parents. Crossover adopts parental-pairs of selection step and helps them to produce offspring. While, the mutation operator slightly modifies the parental string so as to diversify the generation within a population^27^. The GA process went on continuously until it approached to an approximate optimum solution^28^. The GA operations reiterated several times by varying the input space parameters until the accomplishment of suitable results^19^.

## Theory and calculations

Expressions for the evaluation of uptake capacity of biomass, percentage removal of As(III), reaction kinetics, sorption isotherms, thermodynamic constants and the parameters for packed bed bioreactor have been enlisted in Table 1^21,29,30^.

**Table 1:** List of equations for the calculation of different parameters.

Parameters	Equation/s	Symbols and abbreviations:
**Formulae for calculation of batch biosorption parameters and models**^21^		
Uptake capacity Percentage removal	$q_{t} = \frac{{(C_{i} - Ct) \, \times \, V}}{W}$ $\% {\text{Re}} moval = \frac{{(C_{i} - Ct) \, }}{Ci} \times 100$	q_t_: Uptake capacity (mg/g) at time t *C*_*i*_: Initial metal ion concentration in solution (mg/L) *C*_*t*_: Remaining metal ion concentration in solution (mg/L) V: Volume of solution (L) W: Weight of biosorbent (g)
**Sorption isotherms**		
Langmuir sorption isotherm:	${\text{C}}_{{\text{e}}} /q_{e} = 1/Q^{o} b + {\text{C}}_{{\text{e}}} /Q^{o}$	*C*_*e*_: Equilibrium concentration of solute in solution (mg/L) *q*_*e*_: Uptake capacity at equilibrium (mg/g) *b*: Langmuir constant related to free sorption energy (L/mg) *Q*^*o*^: Represents sorption capacity of biosorbent (mg/g)
Freundlich sorption Isotherm:	$\log q_{e} = \log K_{F} + 1/n\log C_{e}$	*K*_*F*_: Freundlich constant indicative of uptake capacity (mg/g) n: Freundlich constant, indicates the intensity of sorption
**Thermodynamic studies**		
Gibbs–Helmholtz equation Equilibrium constant Van 't Hoff equation	$\Delta G = - RT\ln Kc$ $K_{c} = C_{Ae} /C_{e}$ $\ln K_{c} = - \Delta H/RT + \Delta S/R$	∆G: Gibbs free energy change (kcal/mol) R: Universal gas constant (8.314 J/(mol K) *K*_*c*_: Equilibrium constant *C*_*Ae*_: Sorbate concentrations over sorbent surface (mg/L) ΔH: Enthalpy change (kcal/mol); ΔS: Change in the entropy (cal/mol/K)
**Sorption kinetics**		
Pseudo first-order model Pseudo second-order model Initial sorption rate (*h*)	$$\log \left( {q_{e} - q_{t} } \right) = \log (q_{e} ) - \frac{{k_{s} }}{2.303}t$$$$\frac{t}{{q_{t} }} = \frac{1}{{k_{2}^{^{\prime}} q_{e}^{2} }} + \frac{1}{{q_{e} }}t$$ $$h = k_{2}^{^{\prime}} q_{e}^{2}$$	*k*_s_: Equilibrium rate constant (min^-1^) *k*_*2*_*’*: Equilibrium rate constant (g/mg/min) *h*: Initial sorption rate (mg/g/min)
**Calculation of fixed bed bioreactor parameters**^30^		
**Breakthrough curve (C**_**t**_**/C**_**o**_** vs. time):** Effluent volume treated up to breakthrough time (*t*_*b*_) and bed exhaustion time (*t*_*e*_) is denoted as *V*_*b*_ and *V*_e_ respectively	$V_{eff} = Q \times t$	*V*_*eff*_: Volume of effluent (mL) *Q*: Volumetric flow rate of solution (mL/min) t: Time [breakthrough time (*t*_*b*_), bed exhaustion time (*t*_*e*_)]
**Total amount of metal ion fed to the bioreactor (X, mg):**	$X =_{{}} \frac{{C_{o} Qt_{e} }}{1000}$	X: Total amount of metal ion fed to the bioreactor (mg) *C*_*0*_: Metal ion concentration in the inlet solution (mg/L) *t*_*e*_: Bed exhaustion time (min)
**Uptake capacity of the biomass in bioreactor (q, mg/g)**	${\text{q }} = \frac{{q_{tot} }}{M}$	M: Weight of biosorbent in bioreactor (gm) *q*_*tot*_: Total quantity of metal adsorbed in the bioreactor *(q*_*tot*_,: calculated from the area under breakthrough curve)
**Elution efficiency (E)**	$E{\text{ (\% )}} = {\text{md/qtot}} \times {100}$	m_d_: Metal mass desorbed (m_d_,: was calculated from the elution curve (C_t_ vs. t))
**Empty bed contact time (EBCT, min)**	$EBCT = \frac{{V_{C} }}{Q} = \frac{{A_{c} Z}}{Q}$	*V*_*c*_: Sorbent volume in the bed (mL) *A*_*c*_: Bioreactor cross-sectional area (cm^2^) *Z*: Bed height (cm)
**Sorbent usage rate (U**_**r**_**, g/L)**	$U_{r} = \frac{M}{{V_{b} }} = \frac{{V_{c} \rho }}{{V_{c} N_{b} }} = \frac{\rho }{{N_{b} }}$	*ρ*: Sorbent density in the bioreactor (g/cm^3^) *N*_*b*_: Bed volumes treated up to breakthrough *V*_*b*_: Volume treated up to breakthrough time (mL)
**Critical bed height (Z**_**o**_**, cm)**	$Z_{o} = \frac{u}{{k_{a} N_{o} }}\ln \left( {\frac{{C_{o} }}{{C_{b} }} - 1} \right)$	*u*: Linear velocity of solution in bioreactor (cm/min) *k*_*a*_: Rate constant in BDST model (L/mg/min) *N*_*o*_: Sorption or uptake capacity of bed (mg/L) *C*_*b*_: Breakthrough concentration of metal ions (mg/L)

## Results and discussion

### Effect of Immobilization biomass growth and As(III) biosorption

FFB and IFB produced in step 1 as described in material and method section were investigated for the effect of immobilization on growth of biomass. After comparison of dry weighs of FFB and IFB, it was revealed that biomass yield was 18% higher for IFB. It was because luffa sponge provided good support and prevented compact pellet formation resulting into better nutrient supply and rapid growth of fungal mycelia over it.

FFB and IFB were then investigated for the effect of immobilization on As(III) biosorption at arbitrarily fixed experimental condition. The comparative biosorption studies have illustrated that the immobilization had increased the As(III) removal by 4.9% and uptake capacity by 0.05 mg/g in case of IFB (Supplementary data: Table S1). It occurred due to that biomass, after immobilization, becomes less compact as compared to FFB pellets and so the effective surface area for sorption increases. In addition to this, the reduced compactness also increases the accessibility of binding sites over fungal surface which results in higher uptake of the metal^31^.

### Effect of chemical-modification on As(III) biosorption

The chemo-tailored FFB and chemo-tailored IFB produced in *step 2* (Supplementary data: Figure S2) were investigated for analysing the effect of chemo-tailoring on percentage As(III) removal and uptake capacity of biomass by keeping unmodified biomasses (FFB and IFB) as control. The experimental conditions were fixed arbitrarily. As in results, removal of As(III) and uptake capacity was increased by 6.2% and 0.07 mg/g in case of chemo-tailored FFB, whereas these were 6.8% and 0.07 mg/ in case of chemo-tailored IFB respectively (Supplementary data: Table S1). The increment in uptake capacity of chemo-tailored biomasses might be because of generation of additional binding sites over fungal surfaces by acid mediated cleaning of the surface by replacing the pre-bounded ionic species with surface groups^32^. Moreover, mild acid hydrolysis of chitin present in fungal cell wall produces purer amino sugars (D-glucosamine) onto the fungal biomass surface which increases the sorption of metal anions, as mentioned in different reports^21,32^.

### Quantification of impregnated iron and its effect on As(III) biosorption

Figure S2 (Supplementary data) shows the impregnation efficiency of iron on CFB and IIFB as function of pH. From the results, it was clear that impregnated quantity of iron onto the biomasses was strongly dependent on the initial solution pH. The highest impregnation efficiencies of CFB and IIFB were 10.1 and 10.5 g/kg of biomass, respectively at pH 2. Further rise in pH from 2 to 9, the impregnation efficiency abruptly reduced in both the cases. This might be due to high solubility and high diffusion efficiency of soluble iron from bulk solution to biomass surface at lower pH^22^. Higher impregnation efficiency of IIFB was due to its low compactness as compared to CFB, which results in better diffusion of soluble iron on to the biomass surface.

The effect of iron impregnation over As(III) biosorption was investigated by comparing the percentage As(III) removal and uptake capacities of iron impregnated biomasses (CFB and IIFB) with that of their respective chemo-tailored biomasses at arbitrarily fixed experimental conditions. The As(III) removal as well as uptake capacities were found to be increased by 32.7% and 0.32 mg/g in case of CFB, whereas those were 33.0% and 0.33 mg/g in case of IIFB as compared to that of their respective chemo-tailored biomasses respectively (Supplementary data: Table S1).

### Effect of pH and sorption mechanism

**Figure 2:**
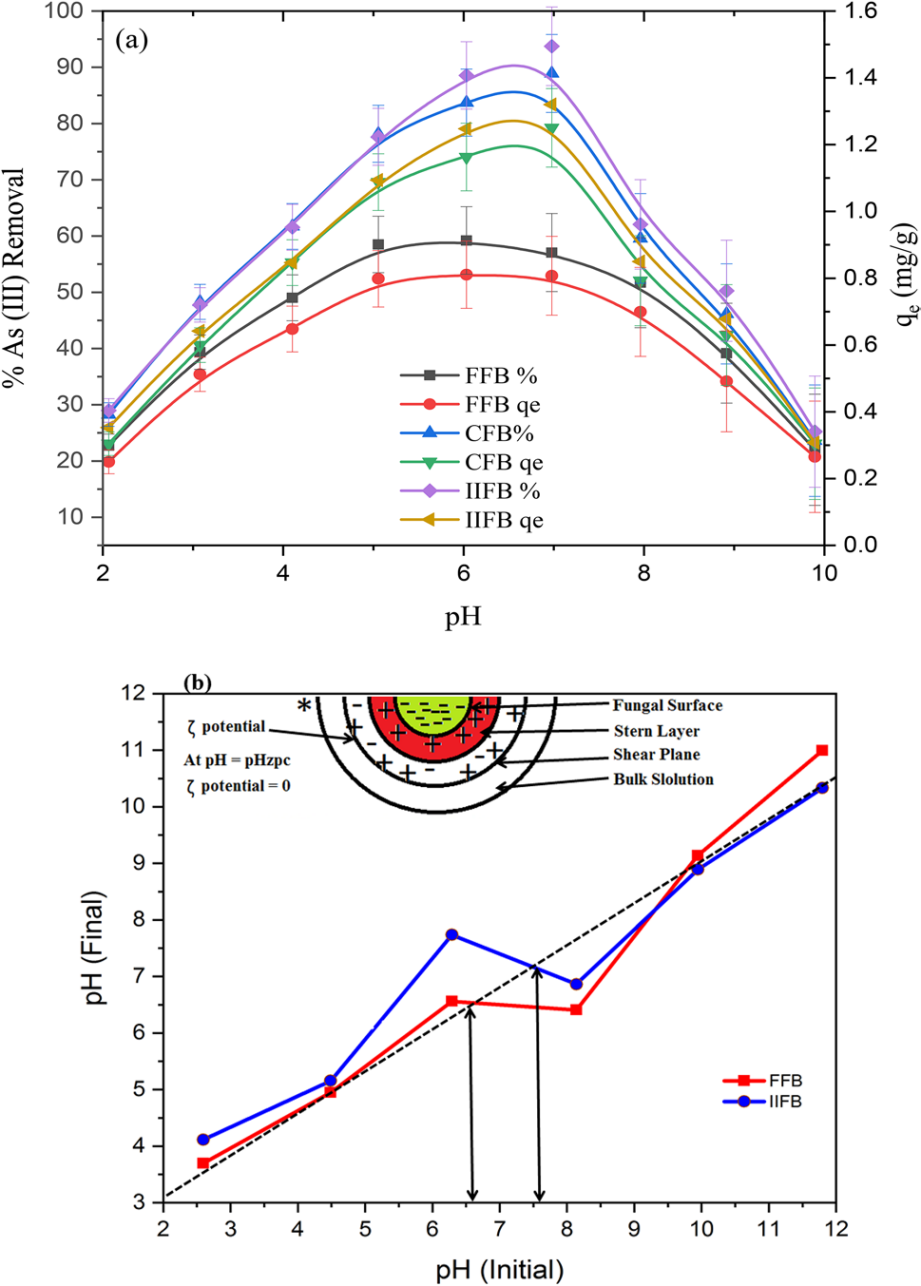
(**a**) Effect of pH on As(III) biosorption over FFB, CFB and IIFB; (**b**) Initial pH vs final pH plot for determination of pH_ZPC_ of FFB and IIFB by solid addition method.

The effect of pH on the biosorption of As(III) was observed by varying pH from 1.0 to 10.0 and maintaining other factors constant at; temperature at 40 °C, initial As(III) concentration of 1.0 mg/L, biomass dose 0.7 g/L and contact time of 135 min. The results thus obtained represented in Fig. 2a showed that the optimum pH for maximum percentage removal and maximum uptake of As(III) for FFB was 6.0 and for CFB and IIFB was 7.0.

Figure 2a represents that FFB showed maximum percentage removal 58.1% and maximum uptake of 0.83 mg/g at pH 6.0, whereas CFB and IIFB represent maximum percentage removal of 87.5% and 92.4% along with maximum uptake 1.25 and 1.32 mg/g respectively at pH 7.0. It was also found that in these cases, the percentage removal increased initially with rising pH from 1.0 to optimum value, thereafter it was decreased quickly with rise in pH from optimum to 10.0. This profile can be clarified through the aqueous chemistry of As(III), non-electrostatic exchange mechanism and zero point charge (pHzpc) of biomass. It has been reported in aqueous chemistry of arsenic that, As(III) exists in two forms, non-ionic ‘H_3_AsO_3_^0^’ and anionic ‘H_2_AsO_3_^−^’ at pH range 3–11, as described in following equation (Eq. 5)^33^.

5 $${\text{H}}_{{3}} {\text{AsO}}_{3}^{0} \leftrightarrow {\text{H}}_{{2}} {\text{AsO}}_{3}^{ - } + {\text{H}}^{ + }$$

Both of these As(III) species are found to be sorbed on the surface of sorbent through non electrostatic exchange mechanism, wherein hydroxyl ions act as an exchangeable legend for H_3_AsO_3_^0^ and water molecule for H_2_AsO_3_^-^. Both of the species ultimately bounded with amino and hydroxyl groups present over the surface of biomass. Similar mechanism has already been established by other workers also^33,34^.

Further to justify that non electrostatic exchange mechanism was involved during sorption of As(III), experiments were conducted to measure the zero point charge of FFB and IIFB with the help of solid addition method^25^. The results of zero-point charge (Fig. 2b) showed that pHzpc of FFB was 6.3 and pHzpc of CFB was 7.2, which were very close to optimum pH for maximum sorption. These results indicated that neutral surface favors maximum sorption. Zero-point charge also explained that with increase in pH from 1.0 to optimum (6.0 or 7.0) the surface protonation decreases and this decrease in protonation leads towards lowering of surface charge, which will support non-electrostatic legend exchange interaction between sorbate and sorbent. Further increase in pH sifted from optimum to alkaline range, the biomass surface attains repulsive negative charge leading to decrease in sorption. In addition to this, at alkaline pH hydroxyl anions becomes dominant, which will compete with anionic species of As(III) (H_2_AsO_3_) resulting towards lower uptake of As(III)^35^.

**Figure 3:**
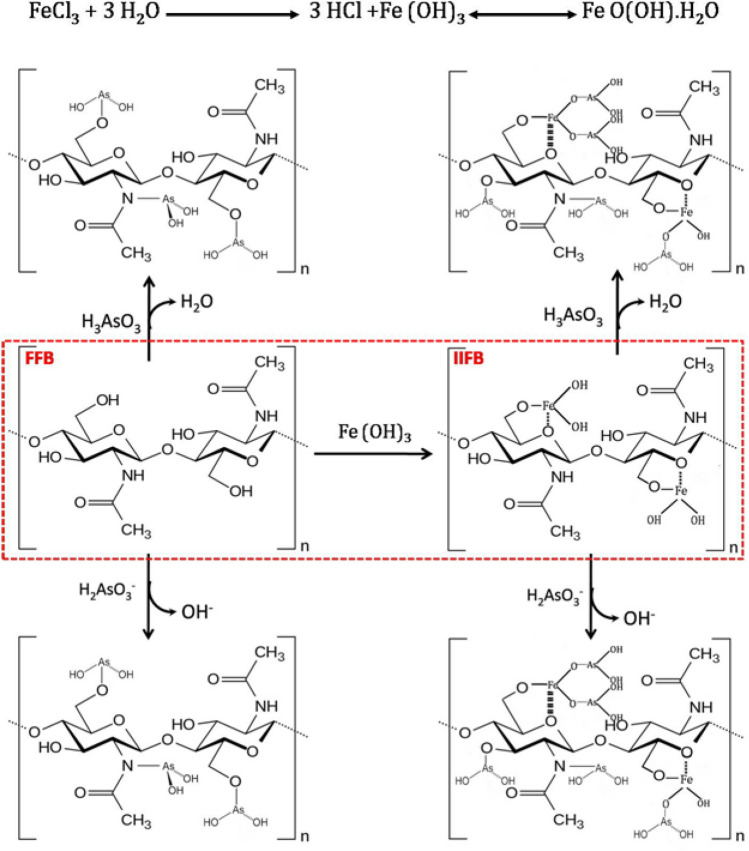
Proposed mechanism of As(III) biosorption over raw and iron impregnated fungal biomass (FFB and IIFB).

It is worth mentioning that major component of fungal cell walls is chitin, which plays a major role during the sorption process. Probable non-electrostatic ligand exchange mechanism for arsenic sorption onto the virgin and iron impregnated fungal biomass surface (chitin) is also illustrated by schematic diagram as shown in Fig. 3. FTIR study of unmodified biomass, modified biomass prior and after the sorption, also strengthens the proposed mechanism.

### Effect of biomass dose

The effect of FFB, CFB and IIFB biomass dose on As(III) uptake was examined at different biomass doses varying from 0.1 to 1.0 g/L and maintaining other factors constant at; 1.0 mg/L initial metal ion concentration, 40 °C of temperature, contact time of 135 min and pH 6 for FFB while pH 7 for CFB and IIFB.

**Figure 4:**
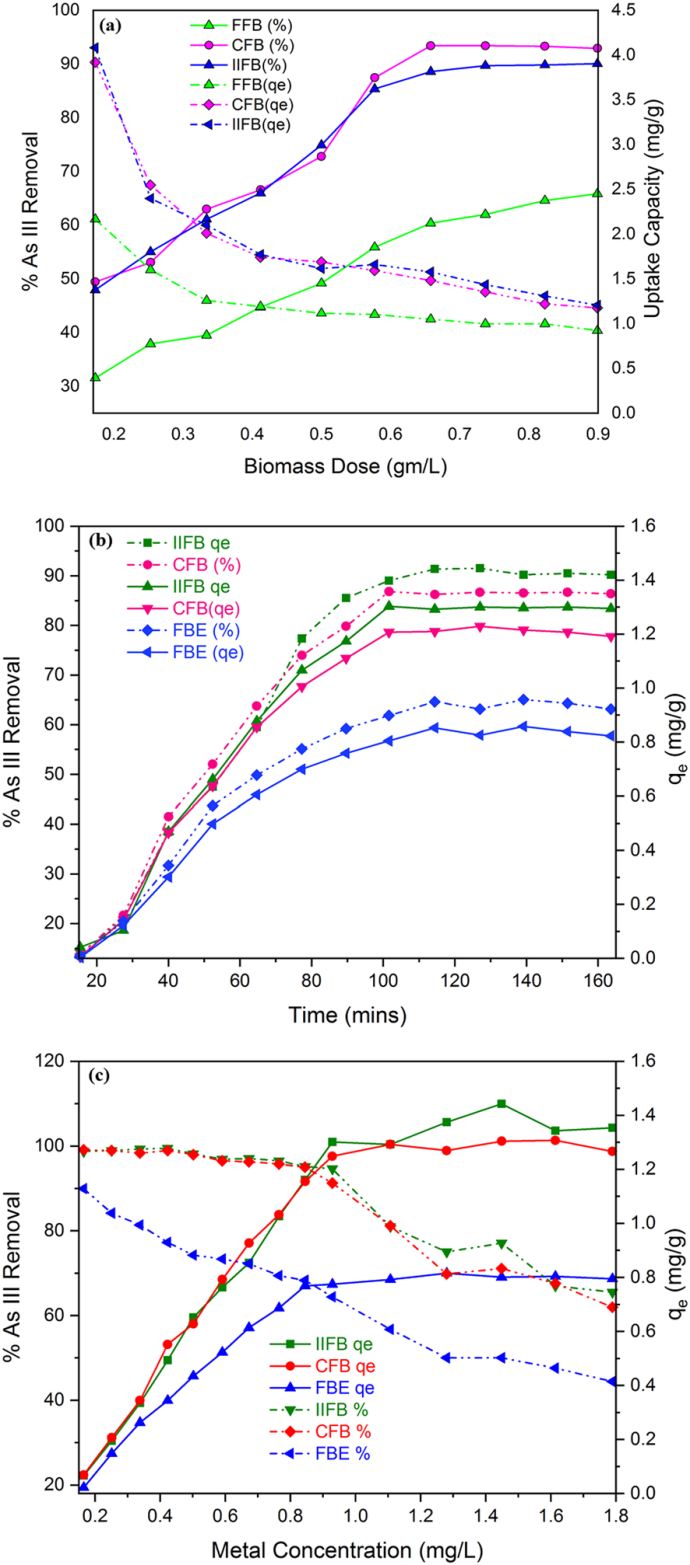
(**a**) Effect of biomass dose on the biosorption of As(III) over FFB, CFB and IIFB. (**b**) Effect of time on As(III) biosorption over FFB, CFB and IIFB. (**c**) Effect of initial metal concentration on As(III) percentage removal and sorption capacity of FFB, CFB and IIFB biomasses.

Results shown in Fig. 4a clearly depicts all forms of biomass i.e. FFB, CFB and IIFB that when biomass dose increased from 0.1 g/L to 1.0 g/L, the uptake capacity of FFB, CFB and IIFB decrease from 2.05 to 0.62 mg/L, 4.0 to 0.91 mg/g and 4.2 to 0.95 mg/g respectively. While percentage metal removal was increased from 20.5% to 58.1% for FFB, 39.6% to 87.5% for CFB and from 42.0% to 92.4% for IIFB respectively.

This can be explained that the uptake capacity of biomass depends upon metal-to-binding sites ratio. As the biomass dose increases, aforesaid ratio decreases leading in depletion of uptake capacity of biomass. However, at higher biomass dosage the available metal ions becomes inadequate to cover all the vacant sites entirely present on the biomass surface, as a result fewer metal ion gets adsorbed at per unit weight of biomass finally lowering the uptake capacity of biomass. In contrast, the percentage As(III) removal increases with increase in biomass dose because the efficiency of As(III) removal shows direct correlation with figure of binding sites i.e. greater the number of available binding sites, greater is the percentage removal. However, at excessive biomass dose, the adsorption is regulated by equilibrium and not by available binding sites. Therefore, beyond a certain limit of biomass dose, the percentage removal curve attains a plateau which represents the optimum biomass dose, in this study it was found to be 0.7 g/L.

### Effect of contact time

The time of equilibrium for current sorption system was evaluated by measuring the residual As(III) concentration in solution until equilibrium achieved i.e. up to accomplishment of constant metal concentration in solution. These experiments were performed by regular screening of the change in metal ion concentration in the solution at initial pH 6.0 for FFB, 7 for CFB and IIFB, initial metal ion concentration of 1.0 mg/L, biomass dose 0.7 g/L and temperature 40 °C. This was observed from results (Fig. 4b) that primarily rapid sorption occurs and equilibrium is accomplished after 120 min for CFB and IIFB however equilibrium is established after 135 min for the case of FFB. It clearly indicates that the sorption process occurs faster for CFB and IIFB as compared to untreated biomass (FFB). This property of CFB and IIFB is due to availability of greater numbers of binding sites over these biomass surfaces in comparison to FFB and thus iron impregnation improves the affinity of biomasses for arsenic removal.

### Effect of initial metal ion concentration

The effect of variation in metal ion concentration on the percentage As(III) removal and uptake capacity of biomass was examined by experimenting with initial metal ion concentration ranging at 0.1 to 2.0 mg/L by keeping other parameters at their optima.

Figure 4c illustrates that at very low metal ion concentrations the As(III) removal was 100% whereas the uptake capacity of biomass was minimum at corresponding concentrations. In contrast to this, as initial metal ion concentration increased, the percentage removal progressively decreased while uptake capacity of biomass augmented to maxima attaining a state of equilibrium. Further increase in metal ion concentration did not show effect on uptake capacity since at optima all the binding sites present on biomass surface are fully saturated with metal ions and having no availability for excess metal ions. The optimum value of metal ion concentration was found to be 0.9 mg/L for FFB and 1.0 mg/g for CFB and IIFB, beyond which the uptake capacity of biomass did not increase. It clearly illustrated that numeric value of binding sites on FFB was lower than that of CFB and IFB, which got saturated at comparatively lower concentration.

### Process parameter optimization

#### RSM optimization

For RSM optimization, a 2^4^ full factorial design consisting of 31 experiments was carried out to examine the levels of process parameters for removal of As(III) (Table 2). Based on the experimental outputs of CCD, a polynomial equation was generated showing the correlation of maximum percentage As(III) removal (*Y*) with the process parameters, viz*.* biomass dose (*X*_*1*_), pH (*X*_*2*_), temperature (*X*_*3*_), and Initial As(III) concentration (*X*_*4*_). The quadratic equation of RSM model can be given as below:

6 $$\begin{gathered} Y = 94.00 + 9.87X_{1} + 0.017X_{2} + 3.57X_{3} - 5.98X_{4} - 8.68X_{1}^{2} - 22.35X_{2}^{2} - 4.3X_{3}^{2} - 7.23X_{4}^{2} \hfill \\ - 1.85X_{1} X_{2} + 5.75X_{1} X_{3} - 2.22X_{1} X_{4} - 0.53X_{2} X_{3} + 0.33X_{2} X_{4} - 1.3X_{3} X_{4} \hfill \\ \end{gathered}$$

**Table 2:** Full factorial CCD of 31 trials with experimental and model based predicted results.

Biomass dose (*X*_1_)	Ph (*X*_2_)	Temperature (*X*_3_)	Initial As(III) concentration (*X*_4_)	% Removal of As(III)		
				Experimental	Predicted % removal	
					Regression	ANN
**Dataset used for model training**						
0.7	6	35	0.7	88.3	89.9	88.6
0.4	4	40	0.4	59.4	60.6	59.3
0.7	6	35	0.7	89.4	91.9	89.7
1.0	4	30	1.0	64.2	67.7	64.2
1.0	4	40	1.0	63.8	65.6	63.2
1.0	8	30	0.4	53.8	58.7	53.4
0.7	10	35	0.7	58.9	57.1	57.7
1.3	6	35	0.7	76.4	73.3	75.9
0.7	6	25	0.7	63.7	58.5	62.8
0.7	6	35	0.7	92.6	89.9	93.1
1.0	8	40	0.4	78.8	77.9	78.3
0.7	6	35	0.7	91.1	88.3	92.3
0.7	6	35	0.7	90.4	91.1	89.8
0.1	6	35	0.7	75.4	74.3	74.5
0.4	8	30	0.4	59.9	57.2	59.2
0.4	4	40	1.0	67.8	66.2	68.2
0.7	6	45	0.7	71.9	73.5	71.7
0.4	8	30	1.0	69.9	72.6	68.8
0.7	6	35	0.7	90.9	89.8	91.2
0.7	6	35	1.3	94.8	91.8	96.1
0.7	6	35	0.7	87.1	89.7	87.0
0.4	4	30	0.4	59.3	61.6	59.1
1.0	8	30	1.0	66.8	66.8	65.2
1.0	4	40	0.4	64.7	68.4	65.3
**Dataset used for model testing**						
0.4	8	40	1.0	83.4	84.4	84.1
1.0	8	40	1.0	80.6	81.7	80.1
0.7	2	35	0.7	46.2	43.4	47.8
0.4	4	30	1.0	69.2	71.3	70.1
0.4	8	40	0.4	75.4	75.4	75.2
0.7	6	35	0.1	79.8	78.3	78.4
1.0	4	30	0.4	63.1	66.2	63.4

The predicted maximum of the metal removal by RSM was 95.2%, where *X*_1_ = 0.7 g/L, *X*_2_ = 6.62, *X*_3_ = 39 °C, *X*_4_ = 1.1 mg/L. Based on statistical data analysis, the value of *R*^*2*^ and adjusted *R*^*2*^ achieved 0.9656 and 0.9318, respectively. However, the mean percentage error was 7.23. Under the optimized conditions, the removal of As(III) found experimentally was 94.3 ± 0.2%, which was in accordance with the predicted RSM model.

#### The hybrid ANN-GA based model

A neural network topology was designed for current biosorption system in order to refine the RSM optimized parameters and to represent the nonlinearities of the fitness function in a much better way by using ANN toolbox of MATLAB software (Version 7.0, Mathworks, Inc., MA, USA). The model was built with four input (viz*.* biomass dose, temperature, pH, and initial As(III) concentration) and one output parameter i.e. percentage As(III) removal by using error back-propagation method. CCD data was partitioned into the training and test set for keeping the network away from over-training and over-parameterization.

**Figure 5:**
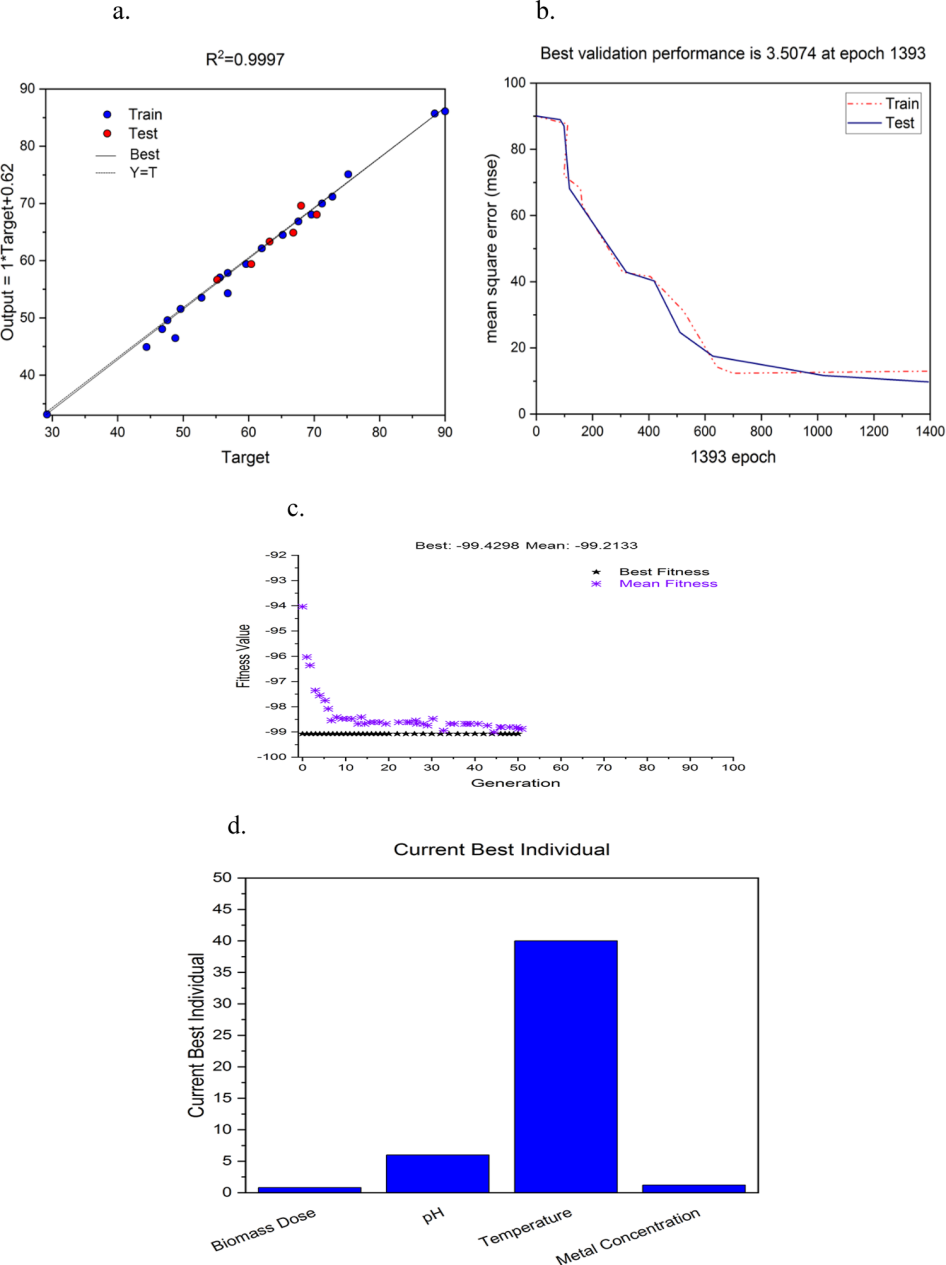
(**a**) Correlation coefficient (*R*^2^) for training and test set of data. (**b**) Mean square error (MSE) of the network during training and test data till final adaptation. (**c**) Best fitness plot showing the progressive performance [%As(III) removal] of GA till the achievement of optimum solution. (**d**) Best experimental individual showing optimum values of all four parameters.

In order to find out the best correlation between experimental and predicted results, the neural network was trained initially with minimum number of neurons (started with five) and further, step by step the number of neurons was varied up to a limit. The network training was also done for different sets of specialized functions such as random initialization, learning rate. The generalization ability of network was determined by assorting the weights generating least test set MSE. The data transfer commands for connecting input to hidden layer and hidden to output layer were utilized as *tansig* and *purelin*, respectively. Some key statements of the algorithm as already described in our other publications^36^ were: %creating ANN: net = newff (minmax[*input matrix*])^1,10^, {'tansig','purelin'}, 'traingdx', 'mse'); %training of ANN: net = train(*net*, *input vector*, *target vector*). %setting the maximum number of training epochs: net.trainParam.epochs = 10,000. %setting the performance of network in terms of ‘mse’: net.performFcn = 'mse' and %setting the ‘mse’: net.trainParam.goal = 0.001. %simulating the output function: Y = sim(*net, input vector of testing dataset*). The model was fully trained after 1393 epochs and the *R*^*2*^ value between the model-predicted and desired As(III) removal with respect to entire data including training and test data was 0.9997 (Fig. 5a,b). The least MSE values for training (E_trn_) and test data set (E_tst_) were 0.023 and 0.031 respectively, which were achieved at the network topology 4-10-1 (i.e. hidden layer with 10 neurons) while learning rate was 0.5. The average prediction error (%) for training and test data set were 2.462 and 3.161, respectively. The lower values of *R*^*2*^, MSE and average prediction error (%) depicted that the ANN-based model had exhibited good approximation and generalization characteristics. Further, results are also comparable about ANN based prediction accuracy over regression model from the Table 2 confirms the better efficacy of ANN as compared to RSM as stated by others^26^.

Finally, genetic algorithm (GA) was implemented to optimize the input space of neural network model with objective of maximization of As(III) removal and it can be exhibited by following expression:

Maximize

7 $$Y = f(x,W);\quad x_{l}^{L} \le x_{l} \le x_{l}^{U} ,\quad l = 1,2, \ldots ,L$$

Here, *f* denotes ANN based objective function; *Y* is maximum As(III) removal; *W* represents equivalent weight vectors; the L-dimensional decision vector, *x,* defines the input parameters (*L* = 4); and $x_{l}^{L}$ and $x_{l}^{U}$ indicates for the lower and upper limits on *x*_*l*_. All four process parameters, during GA optimization process, were confined with the followings constraints as described in experimental data listed in Table 2:

- 0.1 (g/L) ≤ Biomass dose ≤ 1.3 (g/L)
- 2.0 ≤ pH ≤ 10.0
- 25 °C ≤ Temperature ≤ 45 °C
- 0.1 (g/L) ≤ initial As(III) conc. ≤ 1.3 (g/L)

The GA terms in the MATLAB software for the optimization of As(III) removal were fed with following functions: population type: double vector; original population size: 100; elite count: 20; cross over probability: 0.8; crossover function: @crossoversinglepoint; selection function: @selectionroulette; migration direction: forward; mutation function: @mutationgaussian; total generations: 100. For estimation of fitness score of a chromosome (candidate solution) in a random population, following mathematical expression was utilized:

8 $$\varepsilon_{j} = 1 - \frac{1}{{\hat{y}_{pred}^{j} }};j = 1,2,3,......,N$$

where *εj* = fitness score of *j*th candidate solution and $\hat{y}_{\mathrm{pred}}^{j}$= As(III) removal as predicted by model in response to candidate solution.

GA based optimization process was iterated several times by changing the different input space parameters for best global solution. These reiterations at different GA input parameters verified that the entire searching space was surveyed thoroughly to obtain an optimum solution. Reoccurrence of similar optimal solutions for most of the input conditions confirmed that it is a global optimal solution. Finally, GA had resulted the optimum values of biomass dose, metal concentration, pH and temperature, which exhibited maximum As(III) removal. The best fit plot (Fig. 5c) generated during the iterations of GA over generations indicates that resulted solutions were gradually converged towards optimal value. At the ANN-GA optimized condition, As(III) removal was 99.2% at the condition of biomass dose 0.72 g/L, pH 7.31, temperature 42 °C, and initial As(III) concentration 1.1 mg/L (Fig. 5d). This result was authenticated by performing independent experimentation at the set of GA-specified optimum conditions. The percentage As(III) removal accomplished at the above experimental condition was 98.8 ± 0.12% which was in close accordance with the hybrid ANN-GA based prediction.

Results obtained in the present study was quite encouraging as compared to the previous studies done for biosorption of As(III) using different bio sorbents as shown in the Table 3.

**Table 3:** Percentage removal of As(III) using different biosorbents

Biosorbent	Biosorbent type	Maximum percentage removal (%)	References
*Paecilomyces sp.*	Fungal	64.5	^45^
*Aspergillus sp.*	Fungi	53.92	^46^
*Pteris vittata* L	Fern	71.17	^47^
*Acinetobacter, Pseudomonas*	Bacteria	63.35	^48^
*Aneurinibacillus aneurinilyticus*	Bacteria	51.99	^49^

### Isotherm, kinetic and thermodynamic studies

#### Sorption isotherms

**Figure 6:**
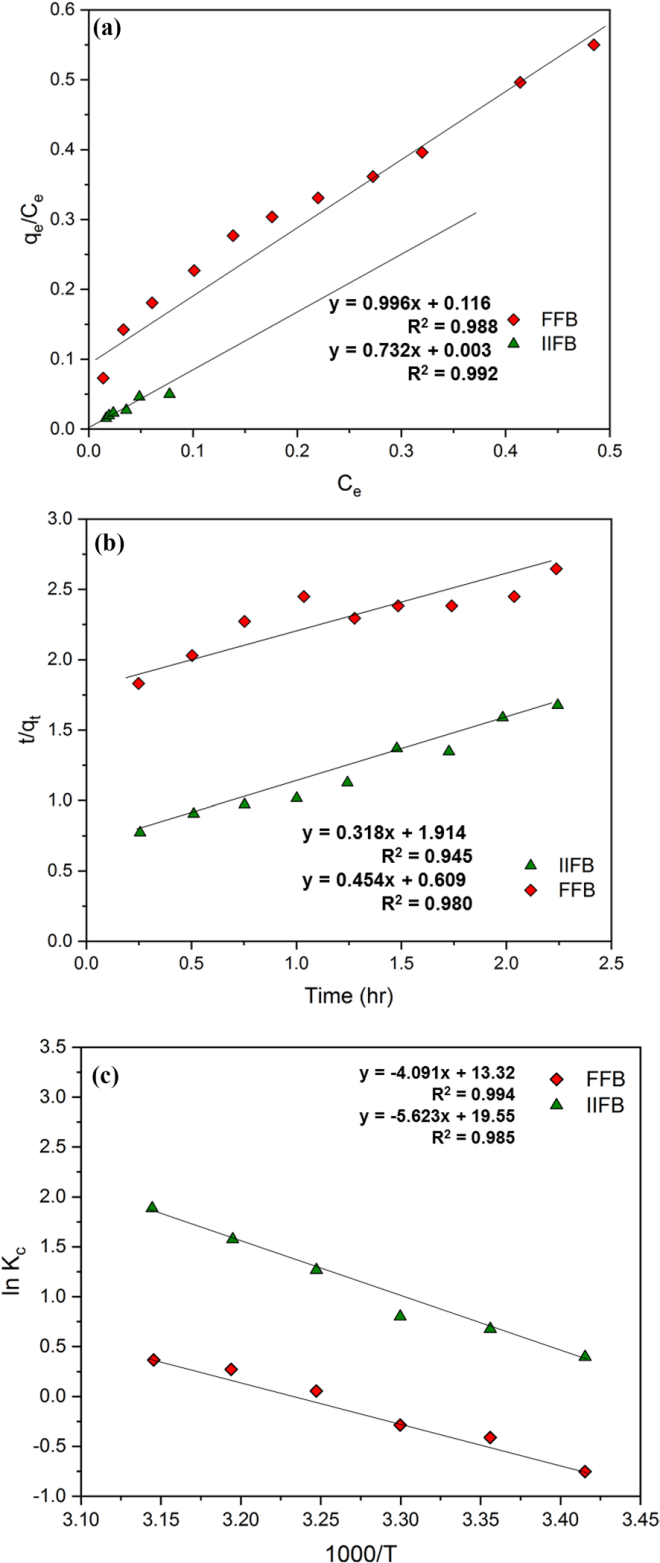
(**a**) Langmuir isotherm plot for As(III) by FFB and IIFB. (**b**) Plot of Pseudo-second-order rate kinetics model for As(III) removal through FFB and IIFB. (**c**) Van’t Hoff plot for As(III) biosorption over FFB and IIFB.

Sorption isotherm illustrates the equilibrium between free and sorbed As(III) over the surface-liquid interface at a given temperature correlating the distribution of As(III) concentration at sorbate-sorbent interface and within aqueous phase with a certain adsorption pattern. The analysis of sorption pattern was performed by using Langmuir and Freundlich isotherm models at initial As(III) concentration varying in range of 0.1 mg/L to 1.1 mg/L while maintaining other parameters at their optimum values. Comparative higher values of correlation coefficients in results clearly indicated that Langmuir isotherm fitted better with the current sorption system (Fig. 6a, Table 4) and it showed that the sorption followed monolayer pattern with sorption energy uniformly distributed over biosorbent surface. Lower values of *b* and higher values of *Q*^*o*^ for IIFB further pointed out that IIFB was a better sorbent system for As(III) sorption as compare to FFB.

**Table 4:** Parameters and model constants for isotherm, kinetic and thermodynamic studies for As(III) biosorption over IIFB, here FFB was taken as control.

Study and models	FFB	IIFB
**Sorption Parameters**		
*Langmuir Isotherm*		
Q˚ (mg/g)	1.004	1.366
b (L/mg)	0.115	0.002
R^2^	0.976	0.992
*Freundlich Isotherm*		
K_F_ (mg/g)	1.412	3.845
1/n	1.937	2.415
R^2^	0.852	0.937
**Kinetic Parameters**		
*Pseudo-first-order model*		
k_s_ (L/min)	2.832	2.367
q_e_ (mg/g)	6.160	3.400
R ^2^	0.745	0.765
*Pseudo-second order*		
k'_2_ (g/mg/min)	0.085	0.338
h (mg/g/min)	0.591	1.642
q_e_ (mg/g)	2.624	2.202
R^2^	0.945	0.980
**Thermodynamic Parameters**		
ΔH (kJ /mol)	34.004	46.749
ΔS (kJ/mol/K)	0.110	0.162
ΔG (kJ/mol) at293K	− 1.615	− 1.008
298 K	− 1.704	− 1.721
303 K	− 1.975	− 2.158
308 K	− 2.485	− 3.392
313 K	− 1.012	− 4.126
318 K	− 0.783	− 3.025

#### Kinetic studies

The studies of sorption kinetics suggest about the rate of solute elimination as well as the residence time of the sorbate at the solid–solution interface. Thus, sorption kinetic studies are pre-requisite for designing a continuous sorption system. These studies were carried out at optimum parameters and sampling was done at regular time interval to measure concentration variation profile of arsenic retained in the system as a function of time. In order to define the kinetic behavior of sorption system, two popular models viz*.* pseudo-first order and pseudo-second order pattern models were followed. Kinetic parameters and correlation coefficient for IIFB and FFB, shown in Table 4, indicates that As(III) sorption over FFB and IIFB follows the pseudo-second order kinetics model (Fig. 6b), which signifies that sorption process is directly proportional to As(III) concentration and the square of the number of free sites over sorbent surface. It was also evident from better fit of pseudo-second order model with experimental data that the rate limiting step in current sorption system was chemisorptive valence force, produced during the exchange or sharing of electrons between sorbent and sorbate, coordination or via complexation^37^.

#### Thermodynamic studies

Thermodynamics of the current biosorption was studied along with varying temperature and constant optimal values of other parameters. Van’t Hoff plots (ln*Kc* vs. 1/*T,* Fig. 6c) were drown and values of correlated thermodynamic parameters were examined using equations from Table 1. The negative magnitude of ∆*G* at all the temperatures reflected the spontaneity of sorption process (Table 4). However, positive enthalpy change (Δ*H*) depicted that the process was endothermic in nature. Furthermore, change in entropy (Δ*S*) with positive sign signified about increasing the degree of freedom of metal ions during sorption process this might be owing to the randomness of the sorption at solid–liquid interface^38^.

#### Removal of As(III) by IIFB discs in packed bed bioreactor

On the basis of performance in batch mode, IIFB was chosen as sorbet for packed bed bioreactor experiments and the ability of IIFB discs to act as a bio-filter was evaluated.

**Figure 7:**
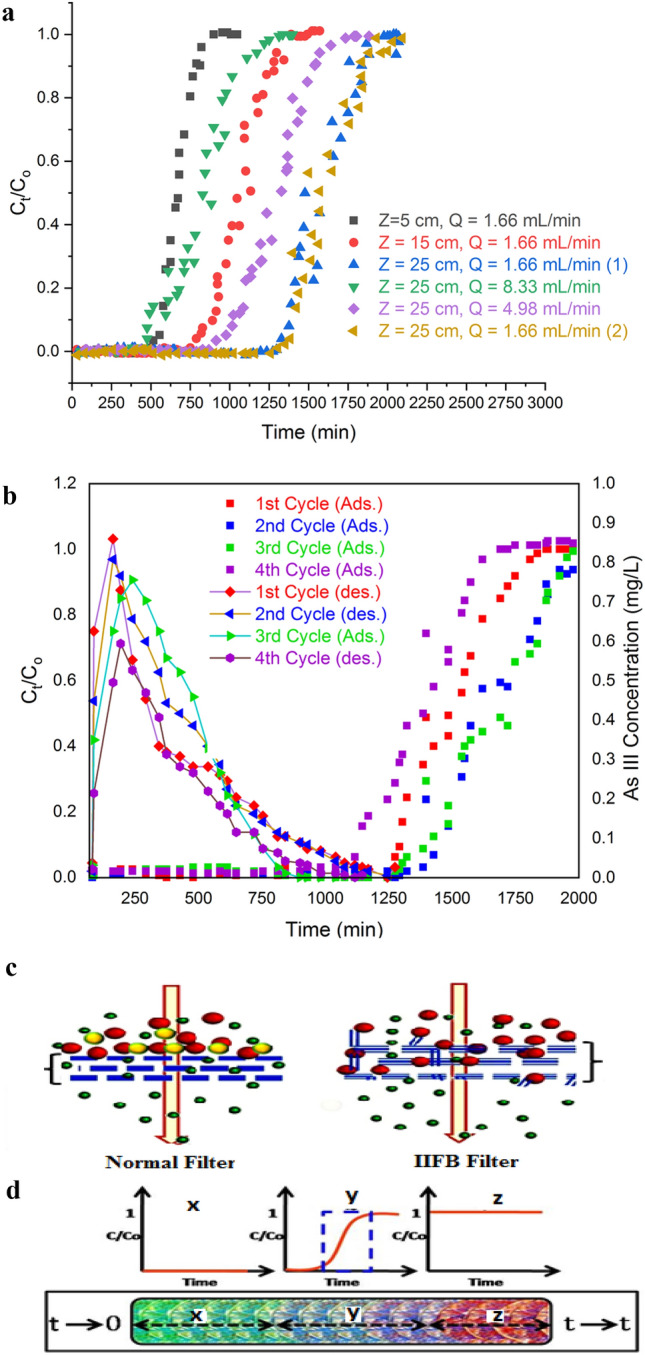
(**a**) Breakthrough curves for As(III) removal in continuous up-flow packed-bed bioreactor at different bed heights (5, 15, 25 cm) of IIFB biosorbent and different flow rates (1.66, 4.98 and 8.33 mL min^−1^) at pH 7 and As(III) inlet concentration of 1.1 mg/L. (**b**) Adsorption and desorption curves for As(III) removal in IIFB packed-bed-bioreactor in successive four cycles. (**c**) Sorption based filtration action of IIFB discs. (**d**) Filter exhaustion profile with respect to time (0 to t) and corresponding sections (x, y and z) of breakthrough curve.

The bioreactor experiments were conducting in two sets: first with varying bed heights i.e. 5, 15, 25 cm and maintaining flow rate constant at 1.66 mL/min whereas second set of experiments with varying flow rate i.e. 1.66, 4.98, 8.30 mL/min and the bed height maintained at 25 cm. Breakthrough curves, Ct/C_0_ vs. time, for both the sets of experiments and calculated parameters for biosorption in bioreactor are presented in Fig. 7a and Table 5 respectively. It has been observed from the results that when bed height was raised from 5 to 25 cm at constant flow rate of 1.66 mL/min, breakthrough time (t_b_) increase from 510 to 1350 min and volume treated up to break through point (V_b_) increase from 846.6 to 2241.0 mL It is explained from the fact that at lesser bed heights axial dispersion phenomena predominated in the mass transfer, resulting into reduction in diffusion of the metal ions i.e., metal ions will not have sufficient time to diffuse within whole sorbent^39^. Thus, increase in bed height minimizes this limiting factor i.e. provides enough time to metal ions to diffuse in to biosorbents mass leading to increase in breakthrough volume of treated water. Whereas results for second set of experiments depicts that t_b_ decrease from 1350 to 250 min and V_b_ decrease from 2241.0 mL to 2075.0 mL when flow rate was increased from 1.66 mL/min to 8.30 mL/min at constant bed height of 25 cm. Moreover, this was also observed that percentage removal increases from 76.40% to 88.23% when bed height was raised from 5 to 25 cm and decrease from 88.23% to 69.45% when flow rate was increased from 1.66 mL/min to 8.30 mL/min. This can be attributed to the fact that when flow rate goes down, the residence time increases which lead to increase percentage removal of metal ions.

**Table 5:** Sorption data for As(III) sorption in up-flow packed-bed bioreactor containing IIFB discs as matrix.

Bed height (cm)	Flow rate (mL/min)	t_b_ (min)	t_e_ (min)	V_b_(mL)	V_e_ (mL)	q_tot_ (mg)	q (mg/g)	EBCT (min)	Percentage Removal	X (mg)	Ur (g/L)
**Process Variables: Constant flow rate and different bed height**											
5	1.66	510	910	0846.6	1510.6	1.15	1.64	9.45	76.40	1.51	0.82
15	1.66	810	1390	1344.6	2307.4	1.82	0.87	28.37	79.19	2.30	1.56
25	1.66	1350	1980	2241.0	3286.8	2.92	0.82	47.28	88.23	3.28	1.53
**Process Variables: Constant flow rate and different bed height**											
25	1.66	1350	1980	2241.0	3286.8	2.92	0.82	47.28	88.23	3.28	1.53
25	4.98	430	1310	2141.4	6523.8	5.11	1.46	15.76	78.41	6.52	1.63
25	8.3	250	1110	2075.0	9246.3	6.39	1.82	9.45	69.45	9.21	1.68

Bed height (cm)	Flow rate (mL/min)	t_b_ (min)	t_e_ (min)	V_b_(mL)	V_e_ (mL)	q_tot_ (mg)	q (mg/g)	EBCT (min)	Percentage Removal	Elution Time (min)	Elution Efficiency (E %)
**Performance evaluation of IIFB matrix in successive 4 adsorption–desorption cycles**											
25	1.66	1350	1980	2241.0	3286.8	2.88	0.82	47.28	87.70	1010	99.2
25	1.66	1250	1860	2075.0	3087.6	2.80	0.80	47.28	84.58	930	98.6
25	1.66	1110	1690	1842.6	2805.4	2.24	0.64	47.28	80.02	880	98.0
25	1.66	1010	1650	1676.6	2739.0	2.17	0.62	47.28	79.30	730	97.4

As in a successful biosorption process, reusability of sorbent is an essential requirement which greatly reduces the process cost and opens the possibility of recovery of sorbate. In present study, the reusability of IIFB discs was evaluated up to four successive adsorption–desorption cycles using 10% NaOH solution as eluting agent. The results have been shown in Fig. 7b and calculated parameters for these adsorption–desorption cycles are summarized in Table 5. Results shows that breakthrough time, uptake capacity, elution time, and percent elution efficiency decrease with each consecutive cycle and sifts from 1350 to 1010 min, 0.82 mg/g to 0.62 mg/g, 1010 min to 730 min, and 99.2% to 97.4% respectively. This might be due to broadened mass-transfer zone with each progressive regeneration cycles^39^.

#### IIFB disc as sorption based bio-filter

Normal filter consists of a filtration matrix, which separates impurities from water by using a fine physical barrier, a chemical process or a biological process. Here, in case of IIFB based filter, a filtration matrix is present in form of packed biomass which successfully blocks the passage of inlet As(III) through a physico-chemical biosorption process (Fig. 7c). The concentration of As(III) present in inlet water stream decreases gradually through the IIFB column since these ions get bounded with the active binding sites present over filtration matrix. A suitable bed height of the column allows complete entrapment with no As(III) present in the outlet stream. Figure 7d schematically represents the filter exhaustion profile at different times and corresponding sections (x, y and z) of breakthrough curve. Section x denotes complete removal, y shows incomplete removal i.e. intermediate stage of exhaustion whereas section z represents no removal and complete exhaustion of the filter bed where recharging required.

#### SEM EDX and FTIR analysis

**Figure 8:**
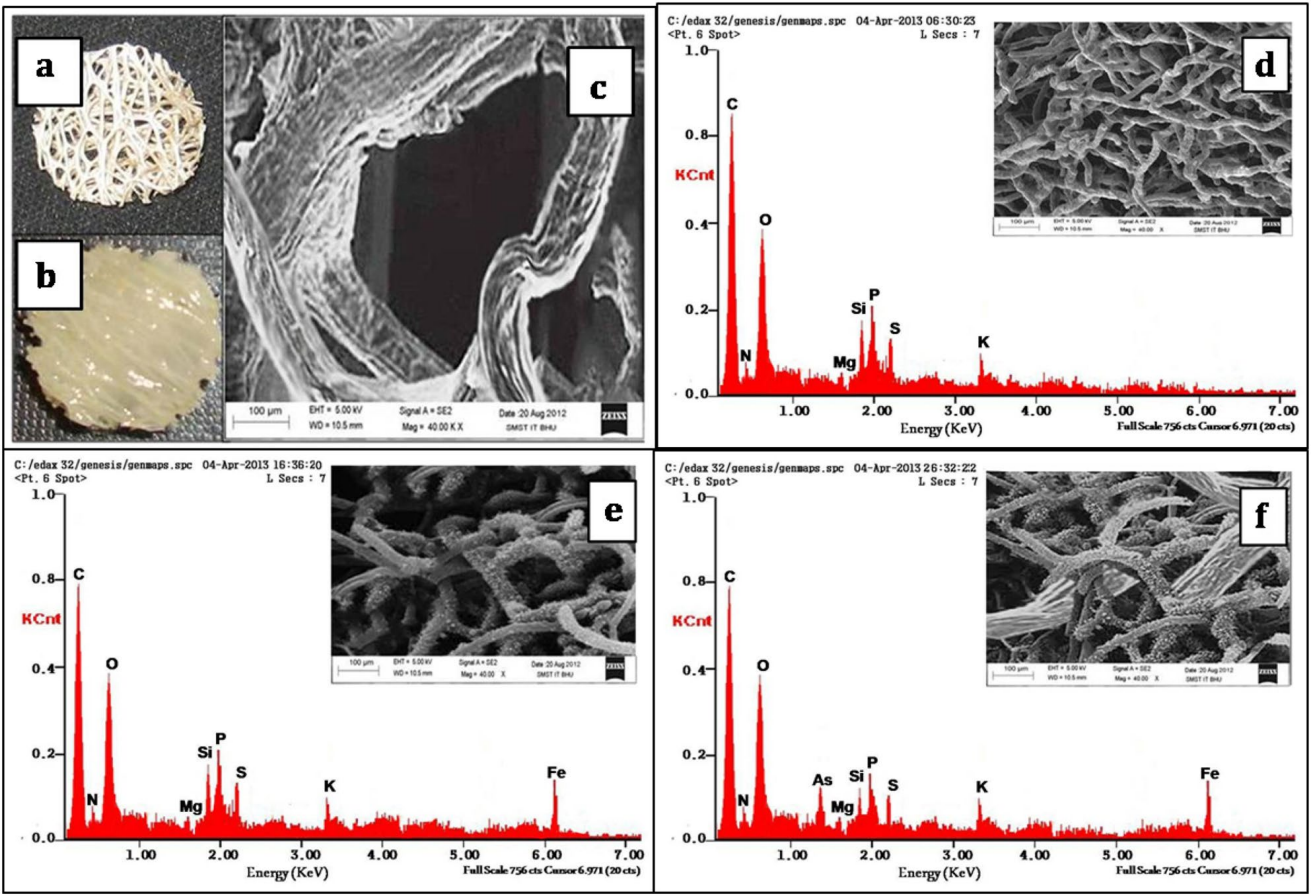
SEM images of raw (FFB; control) and modified (IIFB) biomasses before and after As(III) biosorption and their corresponding EDX spectra. (**a**) Luffa sponge disc. (**b**) Immobilized fungal biomass over luffa sponge disc. (**c**) SEM image showing luffa sponge fiber. (**d**) SEM image of FFB (in inset) and spectra of its EDX analysis. (**e**) SEM image of IIFB (in inset) and corresponding EDX analysis before As(III) biosorption. (**f**) SEM image of IIFB (in inset) and corresponding EDX analysis after As(III) biosorption.

The interwoven fibers within a luffa disc and immobilized fungal biomass over it are clearly visible as in Fig. 8a,b, respectively. SEM images have recorded at the accelerating voltage condition of 5.0kVand beam current 1.0 nA. SEM photograph of interwoven fibers in a luffa sponge was shown in Fig. 8c. Figure 8d–f represents the typical energy dispersive pattern of raw, modified and arsenic loaded modified biomass along with its SEM micrograph. Iron signal in case of modified biomass (CFB, Fig. 8e) confirms the iron impregnation on to the biomass surface whereas arsenic peak present in Fig. 8f denotes the biosorbed As(III) onto the surface of biomass.

**Figure 9:**
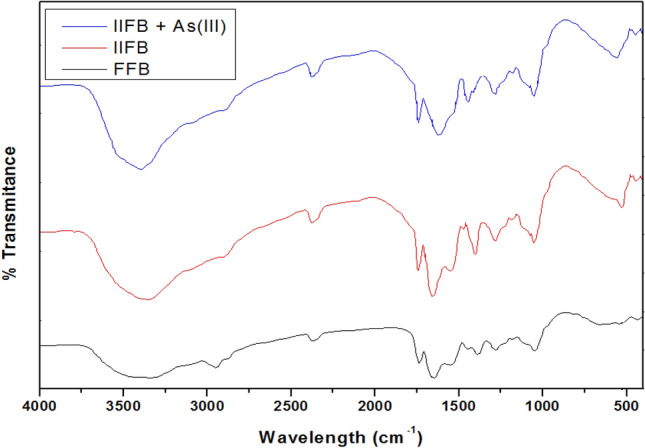
FTIR spectra of untreated fungal biomass (FFB, lower, black line) and Iron-impregnated immobilized fungal biomass (IIFB) before As(III) biosorption (middle, red line) and after As(III) biosorption (upper, blue line).

FTIR spectra for of FFB and IIFB were obtained for the range of 4000–400 cm^−1^, prior after As(III) biosorption, using IR spectrophotometer (Perkin Elmer FTIR-1600) in order to analyze the groups present over the surface of biomass, changes after chemical treatments and groups involve in As(III) sorption. IR spectra shown in Fig. 9 represents different absorption bands, the wide band around 3200–3400 cm^-1^ represents –NH stretching of the protein as well as –OH groups of the glucose and the acetamido group of chitin fraction^38,40^. Absorption peak at 2949.80 cm^−1^ designate the –CH stretching. The absorption peak at 1651.32 cm^−1^ can be a characteristic to amide bonds of protein and the peak at 1550.62 cm^−1^ specifies for primary and secondary amines and amides (N–H bending). The absorbance band around 1404.51 cm^−1^ attributed to N–H stretching, O–H deformation and –CH_2_ scissoring^41^. Peak at 1371.76 cm^−1^ is attributed to sulfamide bonds (S=O). Peaks around 1035.65 cm^−1^ and 1080.38 cm^−1^ represent –CN stretching vibration of chitin and protein fraction. Chemo-tailoring and iron-impregnation, sharpens the –NH stretching and polymeric association band (3200–3400 cm^-1^), which would be owing to emergence of moderately pure amino sugars (D-glucosamines), produced by partial hydrolysis of chitin^42,43^. Along with this, a new strong peak appears at lower frequency around 527 cm^−1^ after iron impregnation, which signifies the iron oxide skeleton^44^. After As(III) adsorption, following siftings in peaks were observed; peak of –NH group sifted from 1651.12 to 1626.99 cm^−1^, peak of O–H deformation shifted from 1404.51 to 1446.37 cm^−1^ and peak of iron oxide skeleton shifted from 527.90 to 555.70 cm^−1^. Which indicate that hydroxyl and amino groups participates in As(III) biosorption. Moreover, the sharpening of band around 3200–3400 cm^−1^ further confirms the involvement of–OH groups in As(III) biosorption.

## Conclusion

**Figure 10:**
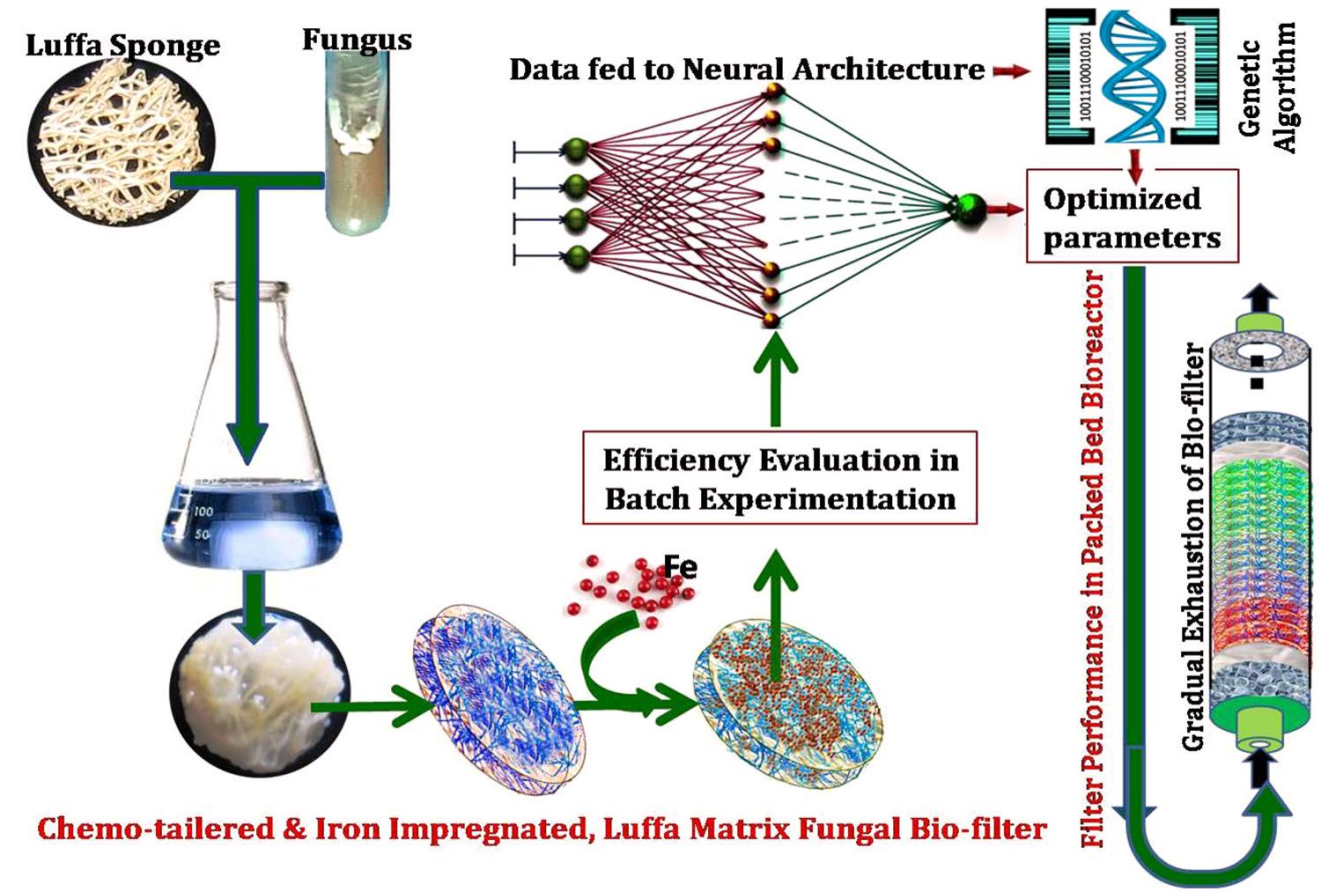
The overall process involved in utilizing luffa sponge as sorption based biofiltration system with enhanced efficiency of As(III) removal in both batch and continuous reactor.

The main aim of this work was effective removal of As(III) from water with the help of a sorption based bio-filtration system. The objective was fulfilled through the development of a biosorbent material by immobilization of dead fungal biomass over luffa sponge. The efficiency of the biosorbent was further improved by chemo-tailoring and iron impregnation. The finally developed biosorbent (i.e. IIFB) was found to be much efficient in batch experiments with 99.2% As(III) removal. The biosorbent, proved to be an excellent filtration biomaterial in a continuous system within a packed-bed bioreactor, because it showed complete removal of As(III) up to 1350 min (≈ 22.5 h) at a bed height of 25.0 cm with a flow rate of 1.66 mL/min and treated 2241.0 mL of water successfully. The system could be termed as a biofilter as it contained microbial biomass attached to a supporting matrix. The overall process involved can be explained with the help of Fig. 10. Though, the biofilter used does not possess metabolic activity but it has filtration ability due to its excellent sorptive properties, it would be better to refer this system as a sorption based fungal biofilter. The involved non-electrostatic legend exchange sorption mechanism for As(III) sorption onto the hydroxyl and amino groups of biosorbent was proposed and established also through pHzpc, FTIR, SEM–EDX analyses and aqueous chemistry of arsenic. Moreover, the sorption characteristics were explored and it was found that sorption followed monolayer pattern in an endothermic way with a spontaneous and pseudo-second order kinetic path. The Biofilter designed could also play significant role in removal of chances of toxicity of other heavy metals in the water as well as As(III) which might be present in Municipal Solid Waste Compost (MSWC) prepared from landfill sites.

## Supplementary Information

Supplementary Information.

## Data Availability

All data generated or analysed during this study are included in this published article [and its supplementary information files].

## References

[1] Singh P, Saraswat A, Pittman CU, Mlsna T, Mohan D (2020). Sustainable low-concentration arsenite [As(III)] removal in single and multicomponent systems using hybrid iron oxide–biochar nanocomposite adsorbents: A mechanistic study. ACS Omega.

[2] Roy PK, Majumder A, Banerjee G, Roy MB, Pal S, Mazumdar A (2015). Removal of arsenic from drinking water using dual treatment process. Clean Technol. Environ. Policy.

[3] Teh CY, Budiman PM, Shak KPY, Wu TY (2016). Recent advancement of coagulation–flocculation and its application in wastewater treatment. Ind. Eng. Chem. Res..

[4] Song ST, Saman N, Johari K, Ma H (2013). Removal of Hg(II) from aqueous solution by adsorption using rawand chemically modified rice straw as novel adsorbents. Ind. Eng. Chem. Res..

[5] de Freitas GR, da Silva MGC, Vieira MGA (2019). Biosorption technology for removal of toxic metals: A review of commercial biosorbents and patents. Environ. Sci. Pollut. Res..

[6] Naghan DJ, Azari A, Mirzaei N, Velayati A, Tapouk FA, Adabi S, Pirsaheb M, Sharafi K (2015). Parameters effecting on photocatalytic degradation of the phenol from aqueous solutions in the presence of ZnO nanocatalyst under irradiation of UV-C light. Bulg. Chem. Commun..

[7] Azari A, Babaie A, Rezaei-Kalantary R, Esrafili A, Moazzen M, Kakavandi B (2014). Nitrate removal from aqueous solution by carbon nanotubes magnetized with nano zero-valent iron. J. Mazandaran Univ. Med. Sci..

[8] Ahmadi E, Kakavandi B, Azari A, Izanloo H, Gharibi H, Mahvi A, Javid A, Hashemi SY (2016). The performance of mesoporus magnetite zeolite nanocomposite in removing dimethyl phthalate from aquatic environments. Desalin. Water Treat..

[9] Azari A, Mahmoudiand MH, Niarie MH, Dehganifardgh I, Es E, Kianii A, Javid A, Azari A, Fakhri Y, Khaneghah AM (2019). Rapid and efficient ultrasonic assisted adsorption of diethyl phthalate onto Fe^II^Fe_2_^III^O_4_@GO: ANN-GA and RSM-DF modeling, isotherm, kinetic and mechanism study. Microchem. J..

[10] Badi MY, Esrafili A, Pasalari H, Kalantary RR, Ahmadi E, Gholami M, Azari A (2019). Degradation of dimethyl phthalate using persulfate activated by UV and ferrous ions: optimizing operational parameters mechanism and pathway. J. Environ. Health Sci. Eng..

[11] RezaeiKalantary R, JonidiJafari A, Kakavandi B, Nasseri S, Ameri A, Azari A (2014). Adsorption and magnetic separation of lead from synthetic wastewater using carbon/iron oxide nanoparticles composite. J. Mazandaran Univ. Med. Sci..

[12] Azari A, Mesdaghinia A, Ghanizadeh G, Masoumbeigi H, Pirsaheb M, Ghafari HR, Khosravi T, Sharafi K (2016). Which is better for optimizing the biosorption process of lead—Central composite design or the Taguchi technique?. Water. Sci. Technol..

[13] Hashemi, S. Y., Azari, A., Raeesi, M., Yaghmaeian, K. (2021). Application of response surface methodology (RSM) in optimisation of fluoride removal by magnetic chitosan/graphene oxide composite: Kinetics and isotherm study. Int. J. Environ. Anal. Chem. 1–9.

[14] Thirumavalavan M, Lai YL, Lee JF (2011). Fourier transform infrared spectroscopic analysis of fruit peels before and after the adsorption of heavy metal ions from aqueous solution. J. Chem. Eng. Data.

[15] Yin K, Wang Q, Lv M, Chen L (2019). Microorganism remediation strategies towards heavy metals. Chem. Eng. J..

[16] Arora NK, Fatima T, Mishra I, Verma M, Mishra J, Mishra V (2018). Environmental sustainability: Challenges and viable solutions. Environ. Sustain..

[17] Deniz F, Karabulut A (2017). Biosorption of heavy metal ions by chemically modified biomass of coastal seaweed community: Studies on phycoremediation system modeling and design. Ecol. Eng..

[18] Sriharsha DV, Kumar RL, Savitha J (2017). Immobilized fungi on *Luffa cylindrica*: An effective biosorbent for the removal of lead. J. Taiwan Inst. Chem. Eng..

[19] Singh Y, Srivastava SK (2013). Statistical and evolutionary optimization for enhanced production of an anti-leukemic enzyme, L-asparaginase, in a protease-deficient *Bacillus aryabhattai* ITBHU02 isolate from the soil contaminated with hospital waste. Indian J. Exp. Biol..

[20] Joshi S, Bajpai S, Jana S (2020). Application of ANN and RSM on fluoride removal using chemically activated D. sissoo sawdust. Environ. Sci. Pollut. Res..

[21] Verma DK, Ranjan D, Hasan SH, Banik RM (2013). Modified biomass of *Phanerochaete chrysosporium* immobilized on luffa sponge for biosorption of hexavalent chromium. Int. J. Environ. Sci. Technol..

[22] Yang J, Park H, Lee H, Lee S (2009). Removal of Cu(II) by activated carbon impregnated with iron (III). Colloids Surf. A.

[23] Verma L, Siddiqui MA, Singh J, Bhargava RN (2019). As(III) and As (V) removal by using iron impregnated biosorbents derived from waste biomass of *Citrus limmeta* (peel and pulp) from the aqueous solution and ground water. J. Environ. Manag..

[24] Prajapati AK, Mondal MK (2019). Hazardous As(III) removal using nanoporous activated carbon of waste garlic stem as adsorbent: Kinetic and mass transfer mechanisms. Korean J. Chem. Eng..

[25] Bakatula EN, Richard D, Neculita CM, Zagury GJ (2018). Determination of point of zero charge of natural organic materials. Environ. Sci. Pollut. Res..

[26] Samuel OD, Okwu MO (2019). Comparison of response surface methodology (RSM) and artificial neural network (ANN) in modelling of waste coconut oil ethyl esters production. Energy Sources Part A.

[27] Kaya M (2011). The effects of two new crossover operators on genetic algorithm performance. Appl. Soft. Comput..

[28] Mohammadi F, Samaei MR, Azhdarpoor A, Teiri H, Badeenezhad A, Rostami S (2019). Modelling and optimizing pyrene removal from the soil by phytoremediation using response surface methodology, artificial neural networks, and genetic algorithm. Chemosphere.

[29] Majumdar S, Baishya A, Mahanta D (2019). Kinetic and equilibrium modeling of anionic dye adsorption on polyaniline emeraldine salt: Batch and fixed bed column studies. Fibers Polym..

[30] Vijayaraghavan K, Jegan J, Palanivelu K, Velan M (2005). Biosorption of copper, cobalt and nickel by marine green alga *Ulva reticulata* in a packed column. Chemosphere.

[31] Iqbal, M. *et al.* Production of Fungal Biomass Immobilized Loofa Sponge (FBILS)-discs for the Removal of Heavy Metal Ions and Chlorinated Compounds from Aqueous Solution. *Biotechnol. Lett.* **27**, 1319–1323 (2005). 10.1007/s10529-005-0477-y 16215832

[32] Qin H, Hu T, Zhai Y, Lu N, Aliyeva J (2020). The improved methods of heavy metals removal by biosorbents: A review. Environ. Pollut..

[33] Kalaruban M, Loganathan P, Nguyen TV, Nur T, Hasan Johir MA, Nguyen TH, Trinh VM, Vigneswaran S (2019). Iron-impregnated granular activated carbon for arsenic removal: Application to practical column filters. J. Environ. Manag..

[34] Saleh TA, Sarı A, Tuzen M (2016). Chitosan-modified vermiculite for As(III) adsorption from aqueous solution: Equilibrium, thermodynamic and kinetic studies. J. Mol. Liq..

[35] Navarathna CM, Karunanayake AG, Gunatilake SR, Pittman CU, Perez F, Mohan D, Mlsna T (2019). Removal of Arsenic(III) from water using magnetite precipitated onto Douglas fir biochar. J. Environ. Manag..

[36] Mohan S, Singh Y, Verma DK, Hasan SH (2015). Synthesis of CuO nanoparticles through green route using *Citrus limon* juice and its application as nanosorbent for Cr(VI) remediation: Process optimization with RSM and ANN-GA based model. Process. Saf. Environ. Prot..

[37] Zhang J (2019). Physical insights into kinetic models of adsorption. Sep. Purif. Technol..

[38] Samir L, Samira A, Mekatel EH, Djamel N (2018). Adsorption of Cr(VI) on *Stipa tenacissima* L. (Alfa): Characteristics, kinetics and thermodynamic studies. Sep. Sci. Technol..

[39] Ranjan D, Talat M, Hasan SH (2009). Rice polish: An alternative to conventional adsorbents for treating arsenic bearing water by up-flow column method. Ind. Eng. Chem. Res..

[40] Tugarova AV, Scheludko AV, Dyatlova YA, Filip;echeva YA, Kamnev AA (2017). FTIR spectroscopic study of biofilms formed by the rhizobacterium *Azospirillum brasilense* Sp245 and its mutant *Azospirillum brasilense* Sp245.1610. J. Mol. Struct..

[41] Giri AK, Patel RK, Mahapatra SS (2011). Artificial neural network (ANN) approach for modelling of arsenic(III) biosorption from aqueous solution by living cells of *Bacillus cereus* biomass. Chem. Eng. J..

[42] Espinosa-Ortiz EJ, Rene ER, Pakshirajan K, van Hullebusch ED, Lens PN (2016). Fungal pelleted reactors in wastewater treatment: Applications and perspectives. Chem. Eng. J..

[43] Anastopoulos I, Bhatnagar A, Bikiaris DN, Kyzas GZ (2017). Chitin adsorbents for toxic metals: A review. Int. J. Mol. Sci..

[44] Sawood GM, Gupta SK (2020). Kinetic equilibrium and thermodynamic analysis of As(V) removal from aqueous solution using iron impregnated *Azadirachta indica* carbon. Appl. Water Sci..

[45] Ismael AR, Víctor M, Juan F, Martínez-Juárez C-G, de Guadalupe M, Moctezuma-Zárate, (2013). Biosorption of Arsenic(III) from aqueous solutions by modified fungal biomass of *Paecilomyces* sp. Bioinorg. Chem. Appl..

[46] Tanvi DA, Pratam KM, Lohit RT, Vijayalakshmi BK, Devaraja TN, Vasudha M, Ramesh A, Chakra PS, Gayathri D (2020). Biosorption of heavy metal arsenic from Industrial Sewage of Davangere District, Karnataka, India, using indigenous fungal isolates. SN Appl. Sci..

[47] Cantamessa S, Massa N, Gamalero E, Berta G (2020). Phytoremediation of a highly arsenic polluted site, using *Pteris vittata* L. and arbuscular mycorrhizal fungi. Plants.

[48] Guo J, Cheng J, Wang J, Hu S (2021). Simultaneous removal of trivalent arsenic and nitrate using microbial fuel cells. Processes.

[49] Dey U, Chatterjee S, Mondal NK (2016). Isolation and characterization of arsenic-resistant bacteria and possible application in bioremediation. Biotechnol. Rep. (Amst.).

