# Author Correction: ANN-GA based biosorption of As(III) from water through chemo-tailored and iron impregnated fungal biofilter system

**DOI:** 10.1038/s41598-022-17593-2

**Published:** 2022-08-02

**Authors:** A. Tripathi, M. R. Ranjan, D. K. Verma, Y. Singh, S. K. Shukla, Vishnu D. Rajput, Tatiana Minkina, P. K. Mishra, M. C. Garg

**Affiliations:** 1 Amity Institute of Environmental Sciences, Amity University Uttar Pradesh, Noida-125, Gautam Buddha Nagar, U.P. 201303 India; 2 School of Biochemical Engineering, Indian Institute of Technology (Banaras Hindu University), Varanasi, U.P. 221005 India; 3 Department of Transport Science and Technology, School of Engineering and Technology, Central University of Jharkhand, Ranchi, Jharkhand 835222 India; 4 Academy of Biology and Biotechnology, Southern Federal University, Rostov-on-Don, Russia 344090; 5 Department of Chemical Engineering, IIT BHU, Varanasi, U.P. 221005 India

Correction to: *Scientific Reports* 10.1038/s41598-022-14802-w, published online 20 July 2022

The Acknowledgments section in the original version of this Article was incomplete.

“This work was supported by Science and Engineering Research Board (SERB), Department of Science and Technology (DST), Government of India, New Delhi, India (Grant No. EMR/2017/004488). The authors are also thankful for facilities and infrastructure provided by Amity University Uttar Pradesh and technical assistance of School of Biochemical Engineering, Indian Institute of Technology (BHU) Varanasi. Special thanks to, Mr. Reet Ram Verma, a farmer for providing luffa sponge.”

now reads:

“This work was supported by the Science and Engineering Research Board (SERB), Department of Science and Technology (DST), Government of India, New Delhi, India (Grant No. EMR/2017/004488) and Laboratory (Soil Health) of the Southern Federal University with the financial support of the Ministry of Science and Higher Education of the Russian Federation, agreement no. 075-15-2022-1122. The authors are also thankful for facilities and infrastructure provided by Amity University Uttar Pradesh and technical assistance of School of Biochemical Engineering, Indian Institute of Technology (BHU) Varanasi. Special thanks to Mr. Reet Ram Verma, a farmer for providing luffa sponge.”

The original Article has been corrected.

