# Supplementary material for: ANN-GA based biosorption of As(III) from water through chemo-tailored and iron impregnated fungal biofilter system
Source: Sci Rep. 2022 Jul 20;12:12414. doi: 10.1038/s41598-022-14802-w (PMC9300712; doi:10.1038/s41598-022-14802-w)
Supplement: Supplementary file 1 — Supplementary Information. [file 41598_2022_14802_MOESM1_ESM.docx]

**ANN-GA based Biosorption of As(III) from water through Chemo-tailored and Iron Impregnated fungal Biofilter system**

A. Tripathi*^1^, Manju Rawat Ranjan^1^, D. K. Verma^2^, Y. Singh^2^, S.K. Shukla^3^, Vishnu D. Rajput^4^, Tatiana Minkina^4^**,** P.K. Mishra^5^**,** M. C. Garg*^1^

^1^Amity Institute of Environmental Sciences, Amity University Uttar Pradesh, Noida-125

Gautam Buddh Nagar-201313, Uttar Pradesh, India

^2^School of Biochemical Engineering, Indian Institute of Technology

(Banaras Hindu University) Varanasi -221005, (U.P.), India.

^3^Department of Transport Science and Technology, School of Engineering and Technology

Central University of Jharkhand, Ranchi- 835 222, (Jharkhand), India

^4^ Academy of Biology and Biotechnology, Southern Federal University, Rostov-on-Don, 344090, Russia

^5^Department of Chemical Engineering IIT BHU, Varanasi -221005, (U.P.), India

**Figure S1.** Schematic flow-sheet describing comprehensive methodology for the production of different forms of biomasses

***Experiments***

***Experiments***

Inoculated in two sets of flasks

Step 1

***Immobilization***

***Fungus (****Phanerochaete crysosporium****)***

Spore Suspension

**Set 1**

**Set 2**

Only Growth media

Growth media + Luffa Discs

**FFB**

**IFB**

***Experiments***

To investigate,

***Effect of Immobilization on biomass growth and As(III) biosorption***

Step 2

**Chemo-tailored FFB**

**Chemo-tailored IFB**

Chemo-tailoring of FFB & IFB by 0.1 N HCl

To Investigate,

***Effect of chemo-tailoring on As(III) biosorption***

|  |  |
| --- | --- |
|  |  |
|  |  |

(Results: Fig. S2)

Here **FFB** was taken as control

Step 3

Iron impregnation of Chemo-tailored FFB & Chemo-tailored IFB

**CFB**

**IIFB**

***Experiments***

To Investigate,

***Quantification of impregnated iron and its effect on As(III) biosorption***

Step 4

Comparative Biosorption Studies in Batch Mode

(**FFB** was taken as control)

**In these studies; FFB, CFB and IIFB** **represents;**

**FFB** : Only Biomass

**CFB:** Biomass + Chemotailoring & Iron impregnation

**IIFB:** Biomass+ Chemotailoring & iron impregnation + Immobilization

**Figure S2.** Content of impregnated iron on CFB and IIFB prepared at different pHs

**Table S1.** Individual biosorption performance of each type of biomass

| ***Step*** | **Type of Biomass** | **Investigated Effect** | **% Removal*^a^*** | **qe**  **(mg/g)** |
| --- | --- | --- | --- | --- |
| **1** | FFB | Immobilization | 42.5% | 0.42 |
|  | IFB |  | 47.4% | 0.47 |
| **2** | Chemo-tailored FFB | Chemo-tailoring | 48.7% | 0.49 |
|  | Chemo-tailored IFB |  | 54.2% | 0.54 |
| **3** | CFB | Iron Impregnation | 81.2% | 0.81 |
|  | IIFB |  | 87.2% | 0.87 |

*^a^*Arbitrarily fixed experimental conditions: pH 7.0; Temperature 25 °C; Biomass Dose 1.0 g/L; As(III) concentration

1.0 mg/L.
